# An innovative green synthesis approach of chitosan nanoparticles and their inhibitory activity against phytopathogenic *Botrytis cinerea* on strawberry leaves

**DOI:** 10.1038/s41598-022-07073-y

**Published:** 2022-03-03

**Authors:** Noura El-Ahmady El-Naggar, WesamEldin I. A. Saber, Amal M. Zweil, Shimaa I. Bashir

**Affiliations:** 1 Department of Bioprocess Development, Genetic Engineering and Biotechnology Research Institute, City of Scientific Research and Technological Applications (SRTA-City), New Borg El-Arab City, 21934 Alexandria Egypt; 2 Microbial Activity Unit, Department of Microbiology, Soils, Water and Environment Research Institute, Agricultural Research Center, Giza, 12619 Egypt; 3 Plant Biotechnology Department, Genetic Engineering and Biotechnology Research Institute, University of Sadat City, Sadat City, Egypt; 4 Department of Plant Protection and Biomolecular Diagnosis, Arid Land Cultivation Research Institute, City of Scientific Research and Technological Applications (SRTA-City), New Borg El-Arab City, 21934 Alexandria Egypt

**Keywords:** Antifungal agents, Nanoparticles

## Abstract

Green synthesis is a newly emerging field of nanobiotechnology that offers economic and environmental advantages over traditional chemical and physical protocols. Nontoxic, eco-friendly, and biosafe materials are used to implement sustainable processes. The current work proposes a new biological-based strategy for the biosynthesis of chitosan nanoparticles (CNPs) using *Pelargonium graveolens* leaves extract. The bioconversion process of CNPs was maximized using the response surface methodology. The best combination of the tested parameters that maximized the biosynthesis process was the incubation of plant extract with 1.08% chitosan at 50.38 °C for 57.53 min., yielding 9.82 ± 3 mg CNPs/mL. Investigation of CNPs by SEM, TEM, EDXS, zeta potential, FTIR, XRD, TGA, and DSC proved the bioconversion process's success. Furthermore, the antifungal activity of the biosynthesized CNPs was screened against a severe isolate of the phytopathogenic *Botrytis cinerea*. CNPs exerted efficient activity against the fungal growth. On strawberry leaves, 25 mg CNPs/mL reduced the symptoms of gray mold severity down to 3%. The higher concentration of CNPs (50 mg/mL) was found to have a reverse effect on the infected area compared with those of lower concentrations (12.5 and 25 mg CNPs/mL). Therefore, additional work is encouraged to reduce the harmful side effects of elevated CNPs concentrations.

## Introduction

Recent advances in the synthesis of diverse nano-molecules of varied sizes, shapes, and functionalities have established nanotechnology as an essential technology in a variety of disciplines^1^. Thus, nanotechnology has been revolutionizing almost all realms in the life sciences and many high-technology industries^2^. Nanoparticles, in contrast to bulk materials, have a greater specific exterior area and a high reaction activity. Thus, have been demonstrated to impact many living cells, including microbial ones^1^.

The natural chitosan polymer (polyglucosamine (1-4)-2-amino-β-d glucose) originates from the whole or partial deacetylation of chitin and occurs as a linear biopolymer of d-glucosamine and *N*-acetyl glucosamine units^2^. Chitosan is biodegradable, soluble in acidic conditions, and carries a positive charge due to the free amino groups on its polymeric chains^3,4^. A growing interest is being focused on the reformation and application of chitosan, of which, a broad spectrum of antimicrobial properties was reported against several pathogens, such as *Phytophthora infestans*^5^.

Chitosan has long been the preferred material to produce nanoparticles in a variety of applications. Compared to other sources, natural chitosan-based nanoparticles (CNPs) have several beneficial features (replenishability, biocompatibility, biodegradability, nontoxicity, high permeability toward biological membranes, and environmental safety), and are thus preferred for a variety of applications, e.g., antimicrobial activities^2,3,6^.

Contrary to the physical and chemical procedures, green synthesis or phytofabrication of nano-molecules offers several advantages, including economization, fastness, safeness, cleanness, and environmentally acceptable, importantly, the reductive capabilities of the proteins and metabolites of the biological entity can easily convert a metal ion into nanoparticle form^7–9^.

Rose geranium or sweet-scented geranium (*Pelargonium graveolens* L’Her*.*, family: *Geraniaceae*) has been successfully proven to be a nonhazardous and environmentally accepted candidate for the rapid and efficient generation of nanomaterials^7,8,10^. The plant is a perennial, hermaphrodite, evergreen, flowering plant with a shrub-like appearance that can grow as tall as 1.5 m. The strongly rose-scented leaves (Supplementary Figure S1) are deeply incised, velvety, and soft to the touch because of the glandular hairs. The plant has a pale soft pink flower^11–13^. Plant leaf and its oil has several therapeutic applications for the healing of several internal and external diseases^11,14^.

*Botrytis cinerea* is one of the most common and destructive fungal pathogens, infecting more than 200 plant species in subtropical and temperate environments, many of which have very economic significance (e.g., strawberries, grapes, and solanaceous vegetables). *Botrytis cinerea* causes gray mold disease, which is obvious on the surface as gray fluffy mycelium. The symptoms are distributed across plant organs and tissues, including postharvest fruit rot, blossom blight, and leaf blight. *Botrytis cinerea* produces a broad range of low-molecular-weight metabolites that induce an oxidative attack of the tissue, aggregation of free radicals, and hypersensitive cell death of plant cells during cuticle penetration and the development of primary lesions^15,16^. The pathogen is primarily regulated using chemical fungicides, considering an environmental issue and the possibility of developing fungicide resistance, as well^15,17^. Thus, finding an alternative safe controlling procedure, such as CNP, is of great importance.

However, to the best of the authors’ information, no work has been performed on the bioconversion of organic compounds, such as chitosan, into nanoparticles using biological systems. Therefore, *Pelargonium graveolens* was used in the current study because it represents a safe base for the biological preparation of CNPs. The current trial is an alternative economic and eco-friendly procedure designed to cover the chasm of CNP preparation based on biological systems as a novel approach for the phytofabrication of CNPs. The characterization of the biofabricated nanoparticles was itemized, likewise, the antifungal features of the generated CNPs were explored on *B. cinerea* as a fungal model.

## Results

### Primary detection of the generated CNPs

**Figure 1:**
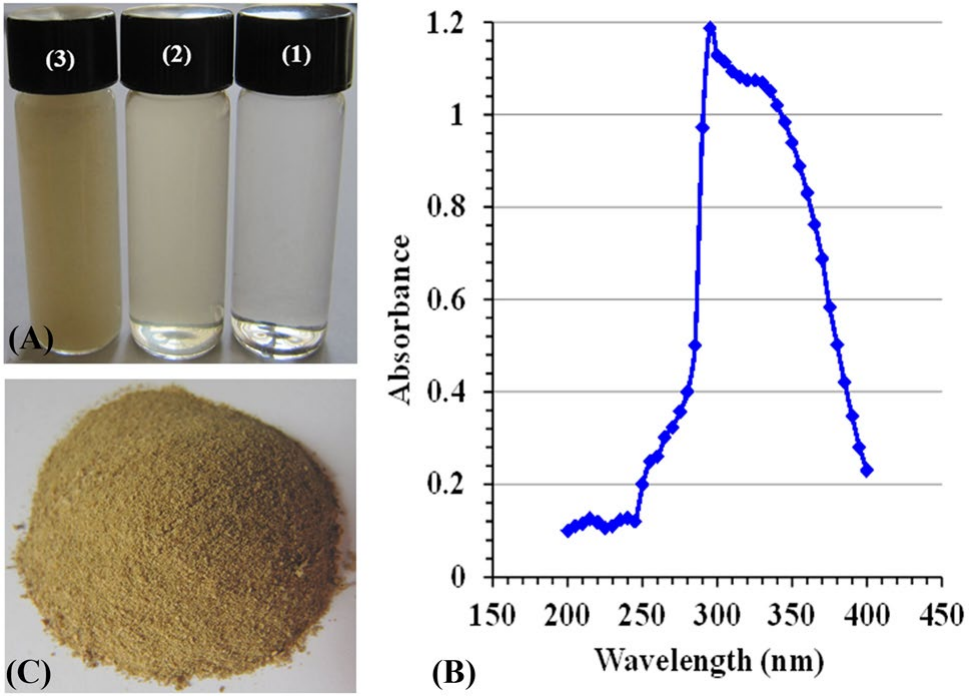
(**A**) Visible observation of chitosan nanoparticle biosynthesis (1, chitosan solution; 2, *Pelargonium graveolens* extract; 3, biosynthesized chitosan nanoparticles), (**B**) UV–Vis spectrum of chitosan nanoparticles, (**C**) dried chitosan nanoparticles biosynthesized using *Pelargonium graveolens* leaf extract.

This study focused on a novel fabrication of CNPs from chitosan solution using *Pelargonium graveolens* leaf extract. Fig. 1A shows three vials containing solutions of chitosan, leaf extract, and biosynthesized chitosan nanoparticles after the biofabrication process. The phytofabrication process of CNPs was monitored and analyzed through a UV/VIS’S spectra range of 200–400 nm (Fig. 1B). The optical properties of the CNP biopolymer materials displayed a single sharp peak, and the maximum absorbance wavelength of CNPs at 295 nm was noticed. The gained CNPs after biosynthesized and drying are depicted in Fig. 1C.

### Modeling the phytofabrication of CNP biosynthesis

To model the bioprocessing conditions of CNP biosynthesis, a statistical modulating design built on response surface methodology was generated. A matrix of Box-Behnken design (BBD), having 17 experimental runs, was used to optimize the phytofabrication process using *P. graveolens* leaf extract. The design matrix, including the real and coded levels of the three variables as well as the experimental and expected values of CNPs and the errors (residuals), are introduced in Table 1. The 17 experimental runs of the phytofabrication process of CNPs showed various responses, ranging from 4.61 to 9.72 mg/mL. the highest value is a center point. However, the actual experimental results of CNPs were found to be in line with the predicted values that were generated based on the equation model.

**Table 1:** Box-Behnken design representing chitosan nanoparticle biosynthesis by *Pelargonium graveolens* as influenced by incubation period (X_1_), initial pH level (X_2_), and chitosan concentration (X_3_).

Std	Run	Incubation period (min)		Temperature (°C)		Chitosan (%)		Chitosan nanoparticles biosynthesis (mg/mL)		Residuals
		Coded	Actual	Coded	Actual	Coded	Actual	Actual	Predicted	
8	1	1	90	0	50	1	1.5	6.62	6.6	0.02
6	2	1	90	0	50	− 1	0.5	4.61	4.51	0.10
2	3	1	90	− 1	40	0	1	5.14	5.23	− 0.09
11	4	0	60	− 1	40	1	1.5	6.19	6.13	0.07
13	5	0	60	0	50	0	1	9.58	9.66	− 0.08
7	6	− 1	30	0	50	1	1.5	6.51	6.61	− 0.10
3	7	− 1	30	1	60	0	1	6.23	6.14	0.09
15	8	0	60	0	50	0	1	9.51	9.66	− 0.15
16	9	0	60	0	50	0	1	9.72	9.66	0.07
14	10	0	60	0	50	0	1	9.76	9.66	0.10
4	11	1	90	1	60	0	1	7.27	7.3	− 0.03
10	12	0	60	1	60	− 1	0.5	4.88	4.95	− 0.07
12	13	0	60	1	60	1	1.5	7.42	7.41	0.01
5	14	− 1	30	0	50	− 1	0.5	5.91	5.93	− 0.02
1	15	− 1	30	− 1	40	0	1	7.85	7.82	0.03
17	16	0	60	0	50	0	1	9.72	9.66	0.06
9	17	0	60	− 1	40	− 1	0.5	5.82	5.83	− 0.01

Variable	Code	− 1	0	1						
Incubation period (min)	A	30	60	90						
Temperature (°C)	B	40	50	60						
Chitosan concentration (%)	C	0.5	1	1.5						

### Model selection

To select the most fitted model, the lack of fit, the sum of squares, and certain model statistics tests were compared for the linear, two-factor interaction, and quadratic models (Table 2). The quadratic model was preferred based on the lower values of probability (*P*) value, standard deviation, and the sum of squares of prediction error (PRESS) and higher values of determination coefficient (R^2^), adjusted-R^2^, and predicted-R^2^. The quadratic model was the most fitted and therefore was selected for the molding process.

**Table 2:** Summary of model fitness for Box-Behnken design results for chitosan nanoparticle biosynthesis using *Pelargonium graveolens* leaf extract as affected by chitosan concentration (%), initial pH level, temperature (°C), and incubation period (min).

Source	Sum of squares	Degrees of freedom	Mean square	*F-*value	*P-*value
**Lack-of-fit tests**					
Linear	48.41	9	5.38	455.22	< 0.0001*
2FI	43.22	6	7.20	609.63	< 0.0001
Quadratic	0.0461	3	0.0154	1.30	0.3896
**The sequential model sum of squares**					
Linear vs Mean	4.91	3	1.64	0.44	0.7288
2FI vis Linear	5.19	3	1.73	0.40	0.7562
Quadratic vis 2FI	43.18	3	14.39	1079.02	< 0.0001*

Source	Standard deviation	R^2^	Adjusted-R^2^	Predicted-R^2^	PRESS
**Model summary statistics**					
Linear	1.93	0.092	− 0.1175	− 0.3632	72.75
2FI	2.08	0.1893	− 0.2972	− 0.9311	103.06
Quadratic	0.12	0.9983	0.9960	0.9848	0.81

*Significant values, PRESS: sum of squares of prediction error, two factors interaction: 2FI.

### Statistical and multiple regression analysis

To discover the significance of the BBD and its factors, both were exposed to the analysis of variance (ANOVA) and multiple regression analysis (Table 3). The overall model generated significant performance with a *P*-value < 0.05. The linear, interaction, and quadratic effects followed the same significant trend. On the other hand, the lack-of-fit error recorded a higher *P*-value > 0.05 that did not allow it to reach the significance threshold. Another, the values of the coefficient of variation (C.V. = 1.60) and adequate precision (58.08) are other evaluating parameters that showed the good performance of the model. All these are indicators of the significance of the model and its involved factors. The general mean and standard deviation values of the investigational runs are 94.95 and 1.53, respectively.

**Table 3:** Analysis of variance for chitosan nanoparticle biosynthesis using *Pelargonium graveolens* leaf extract as affected by initial pH level, the incubation period (min), chitosan concentration (%), and temperature (°C).

Source of variance		Coefficient estimate	Sum of squares	Degrees of freedom	Mean Square	*F*-value	*P*-value
Model	Intercept	9.66	53.28	9	5.92	443.82	< 0.0001
Linear effect	A	− 0.36	1.02	1	1.02	76.66	< 0.0001
	B	0.10	0.08	1	0.08	6.00	0.0442
	C	0.69	3.81	1	3.81	285.56	< 0.0001
Interaction effect	AB	0.94	3.52	1	3.52	263.58	< 0.0001
	AC	0.35	0.497	1	0.497	37.26	0.0005
	BC	0.54	1.18	1	1.18	88.26	< 0.0001
Quadratic effect	A^2^	− 1.60	10.77	1	10.77	807.75	< 0.0001
	B^2^	− 1.43	8.67	1	8.67	649.71	< 0.0001
	C^2^	− 2.14	19.37	1	19.37	1451.93	< 0.0001
Error effect	Lack of fit		0.05	3	0.0154	1.30	0.3896
	Pure error		0.05	4	0.0118		
R^2^		0.9983	Standard deviation			0.12	
Adjusted-R^2^		0.996	Mean			7.22	
Predicted-R^2^		0.9848	Coefficient of variation, %			1.60	
Adequate Precision		58.08					

* Significant values, *F*: Fisher’s function, *P*: Level of significance.

Exploring ANOVA shows the aptness of the various individuals, interactions, and quadratic effects. The three types of R^2^ are adequate to suggest a high significance of both the model and its components. However, the linear, mutual interactions, or quadratic coefficient estimate showed various positive or negative significant responses, being negative for the incubation period and quadratic terms, whereas positive for the rest of the other terms (temperature and initial chitosan concentration) and all the interactions.

### Model adequacy checking

**Figure 2:**
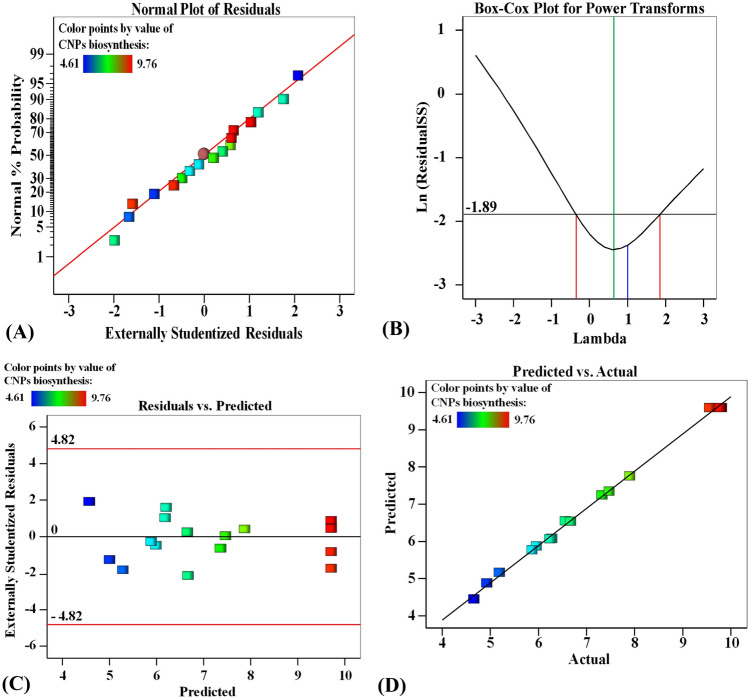
Normal probability plot of residuals (**A**), Box-Cox plot for power transformation (**B**), residuals versus predicted (**C**), and the predicted versus actual (**D**) values of chitosan nanoparticle biosynthesis using *Pelargonium graveolens* leaf extract.

The model adequacy was further checked to approve the appropriateness of the model. Mathematical statics were checked and described. The normal probability plot of the externally studentized residuals (Fig. 2A) displays that data points are concerted thoroughly along the straight line and follow the normal distribution without linearity. In addition, the depiction of the Box-Cox of the power transformation (Fig. 2B) shows that the current lambda (λ) value is equal to one. However, the best λ value (green line) was located between the confidence intervals (two red lines).

**Figure 3:**
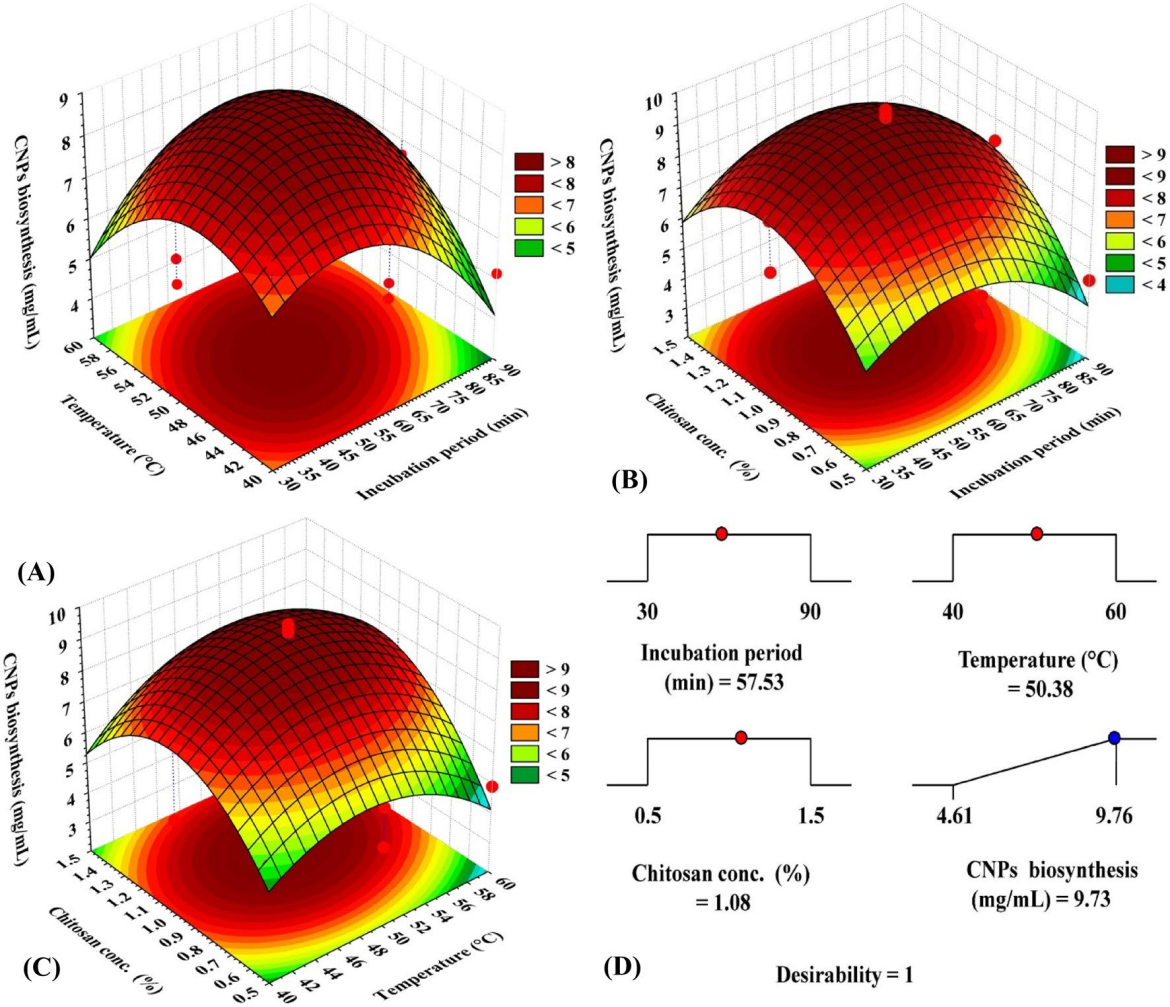
Three-dimensional surface plot for chitosan nanoparticle biosynthesis by *Pelargonium graveolens* leaf extract, showing the interactive effects of each pair of factors, holding the third factor at the center point (**A**–**C**), and the optimization plot displaying the desirability function at the optimum predicted values (**D**) for the maximum chitosan nanoparticle biosynthesis using *Pelargonium graveolens* leaf extract.

Likewise, the values of externally studentized residuals vis predicted values by the model were drawn. The plot (Fig. 2C) shows an equal scatter of the residual data above and below the 0-axis. This pattern is ideal enough to prove the suitability of the BBD model. Similarly, the predicted values were drawn against the trial values to evaluate the aptness of the model (Fig. 2D). Again, the linear regression analysis displays better fitting of the model forecast points that lie nearer to the line of perfect prediction.

### The three-dimensional (3D) surface plot

To explore the mutual influence of each pair of the tested parameters, 3D-surface plots of the simultaneous effect of the three independent factors on CNP biosynthesis using *Pelargonium graveolens* leaf extract were constructed. Fig. 3A–C illustrate two independent factors (at their central points) on the X- and Y-axes against the Z-axis (CNP biosynthesis). The maximum CNP biosynthesis was situated around the central points of temperature, incubation period, and chitosan concentration; out of these ranges, a marked decline in CNP biosynthesis was noticed.

### Experimental validation of the model

The prime aim of the investigational strategy is to find the best-predicted situations for obtaining the maximum CNPs. To find the best-predicted conditions for an extreme response, the desirability function was used, and the best combination of conditions was calculated based on the following prediction equation:

CNP biosynthesis = 9.66 − 0.36 × incubation time + 0.10 × temperature + 0.69 × chitosan concentration + 0.94 incubation time × temperature + 0.35 incubation time × chitosan concentration + 0.54 temperature × chitosan concentration − 1.60 incubation time^2^ − 1.43 temperature^2^ − 2.14 chitosan concentration^2^.

According to the model’s equation, this predicted value was achieved at the tested variables of 57.53 min (incubation period), 50.38 °C, and 1.08% (chitosan concentration). The highest theoretical (predicted) value of CNP biosynthesis was estimated to be 9.73 mg/mL (Fig. 3D). Such optimum points of the tested levels recorded a satisfactory desirability value (1.0).

To verify the green synthesis of CNPs by using *P. graveolens* leaf extract under the optimal predicted conditions, a triplicate set of experiments was carried out, and the experimental results were judged to the predicted values. The actual laboratory value of the green synthesis of CNPs by using *P. graveolens* leaf extract was 9.82 ± 3 mg/mL, verifying a high degree of model precision and confirming the validation of the model under the design matrix.

### Electron microscopy investigation

**Figure 4:**
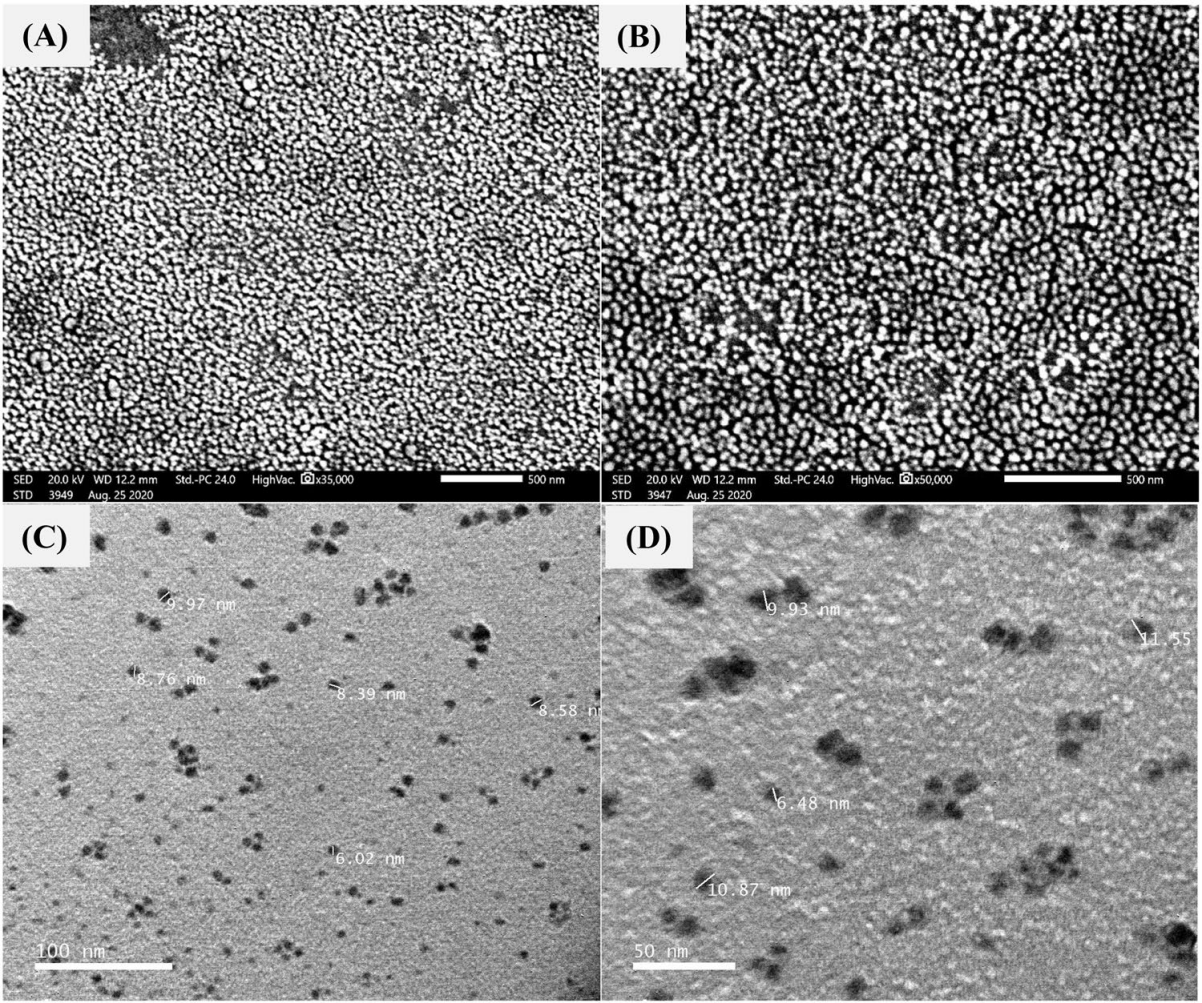
SEM (**A**,**B**) and TEM (**C**,**D**) micrographs of chitosan nanoparticles biosynthesized using *Pelargonium graveolens* leaf extract.

The surface morphological structure was examined using scanning electron microscopy (SEM). The size, morphology, and structure were inspected at various magnification scales (Fig. 4A,B). The SEM image of the morphological construction of CNPs shows spherical-like particles, and their mild agglomerated state revealed a highly porous surface. Furthermore, the size of the nanoparticles also showed uniformity and homogeneity.

Another efficient tool for discovering the morphological structure of CNPs is transmission electron microscopy (TEM), which was performed at various magnification powers (Fig. 4C,D). TEM derives additional details, such as a particle’s aggregation and agglomeration. The two-dimensional **(**2D) TEM image introduced a wider conception than SEM, especially regarding the non-aggregation and lower-agglomeration status of CNPs with a highly porous surface. Furthermore, the CNP size analysis showed a measurement from 6.02 to 10.87 nm. It is of particular importance to note that both SEM and TEM were found to be complementary to the characterization of CNPs.

### Energy-dispersive X-ray spectroscopy (EDXS) analysis

**Figure 5:**
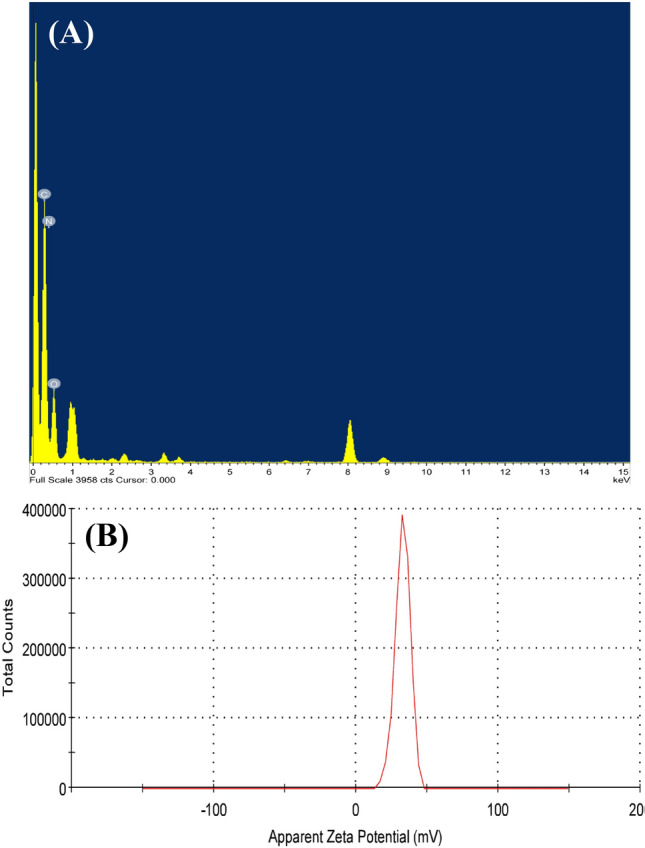
EDXS (**A**) and zeta potential (**B**) analyses of chitosan nanoparticles biosynthesized using *Pelargonium graveolens* leaf extract**.**

For further characterization, CNPs were explored for their elemental compositions using EDXS (Fig. 5A), which can quickly generate information about the kinds of elements, distributions, and concentrations. The EDXS spectra of CNPs confirmed the presence of the four elements that chitosan is composed of hydrogen, carbon, nitrogen, and oxygen. Such a composition represents the main elemental component of chitosan.

### Zeta (ζ) potential analysis

The ζ-potential analysis of the synthesized CNPs was investigated; nevertheless, ζ-potential analysis is often the only available route for the description of double-layer properties. The depicted ζ-potential (Fig. 5B) shows decent stability of the positively charged CNPs with a ζ-potential of + 32.6 ± 5.26 mV at 25 °C. Furthermore, the ζ-potential potential distribution has a single peak, indicating excellent uniformity of CNPs.

### Fourier transform infrared (FTIR) investigation

**Figure 6:**
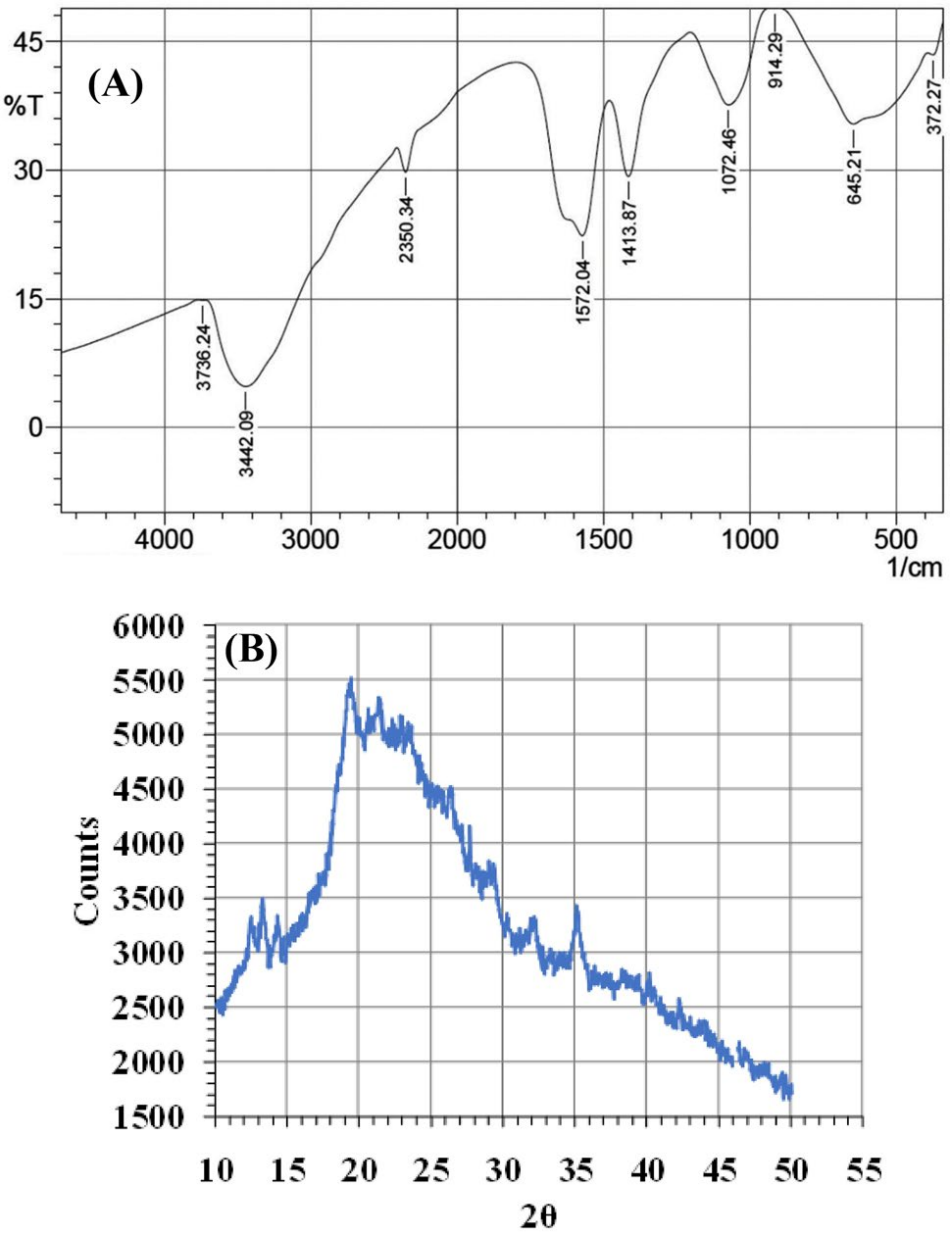
FTIR (**A**) and XRD (**B**) analyses of chitosan nanoparticles biosynthesized using Pelargonium graveolens leaf extract.

CNPs were analyzed using FTIR (Fig. 6A) to explore the biomolecules for the possible occurrence of various functional groups that bind with CNPs due to reduction and stabilization. The detected intense bands were compared with standard values to classify the functional groups. The adsorbent spectra were measured in the range between 4500 and 500 cm^−1^. The characteristics of CNPs were shown by a broad absorption band. The FTIR spectra show absorption bands at 3736, 3442, 2350, 1572, 1413, 1072, 914, and 645 cm^−1^.

### X-ray diffraction (XRD) analysis

The crystallographic structure of CNPs was investigated using XRD (Fig. 6B). After irradiating CNPs with incident X-rays, the intensities and scattering angles of the X-rays that left the CNPs were measured. XRD of CNPs showed three peaks at 2-theta of 13, 19, and 35°.

### Thermal behavior of CNPs

**Figure 7:**
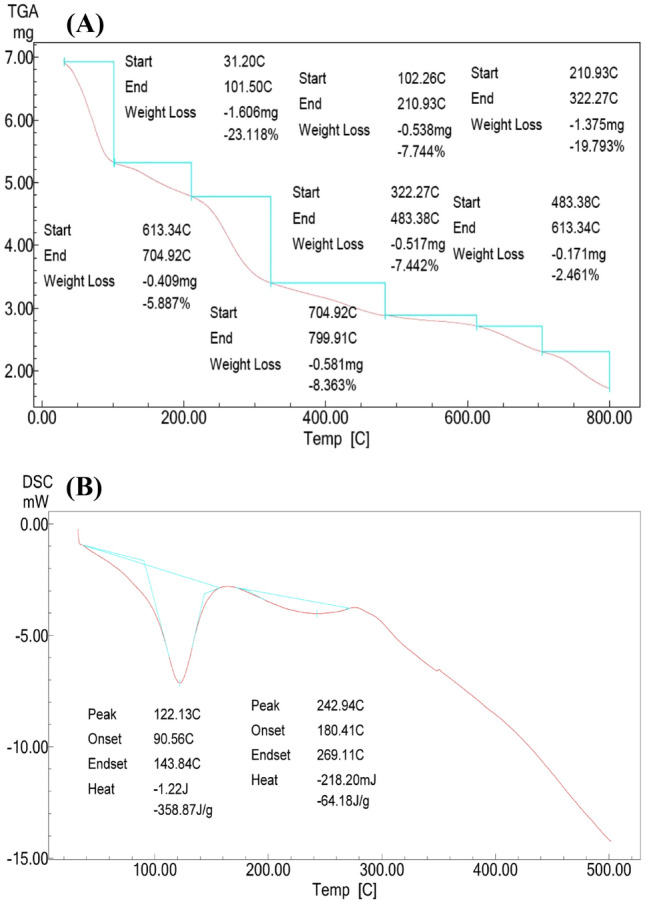
TGA (**A**) and DSC (**B**) analyses of chitosan nanoparticles biosynthesized using leaf extracts of *Pelargonium graveolens*.

The thermal behavior of CNPs was scrutinized by thermogravimetric analysis **(**TGA) in a temperature range from 30 to 800. TGA is a thermal analysis that measures the modifications in physical and chemical features of a material such as CNPs as a function of constant changing heating rate. The change in the mass of CNPs was observed and analyzed with the change in temperature (Fig. 7A). The mass fluctuation of CNPs corresponds to the nature of chemical reactions. At the beginning of heating (31.20–101.5 °C), drying as a function of temperature can easily be seen as a quick initial mass dropping (− 23.118%). Then, the weight loss of CNPs showed multistage decomposition with increasing temperature but with lower weight losses. The lowest weight loss was recorded at a temperature range of 483.38–613.34 °C, which was 2.461%. Although CNPs have reasonable heat constancy, at the last temperature stage (704.92–799.91 °C), the nanoparticles kept a reasonable amount, i.e., ~20%, of their mass.

Investigation of the thermo analytical technique of differential scanning calorimetry (DSC) was applied to CNPs at different heating rates to determine the variation (positive or negative) in the amount of heat flow of CNPs as a function of temperature in the presence of a solvent reference. Both the CNPs and reference were sustained at the same temperature throughout the experiment. Thermo analytical information was gathered to create a phase diagram (Fig. 7B). As the thermodynamic system is changed, the CNPs undergo phase transitions, showing two definite broad endothermic peaks. The first broad endothermic peak was detected between 90 and 143 °C at 122 °C, requiring a heat amount of − 358.87 J/g CNPs. The other broad endothermic peak appeared at 242 °C, requiring − 64.18 J/g CNPs. A single glass transition temperature of the CNPs was found at 180 °C.

### Isolation and identification of *B. cinerea*

Following the isolation and purification trials, the most severe isolate was selected from several isolates based on the pathogenicity test. The colony characteristics of *B. cinerea* SIB-1 were observed on PDA plates, on which the fungus developed and sporulated on the culture medium to cover the majority of the 9-cm plate during six days. The growth pattern on the plates showed the colonies as warty, fluffy, and appressed, grayish-white to light gray or dark gray. The conidia produced on the surface of the medium, in abundance all over the plates, can also be seen as concentric rings.

**Figure 8:**
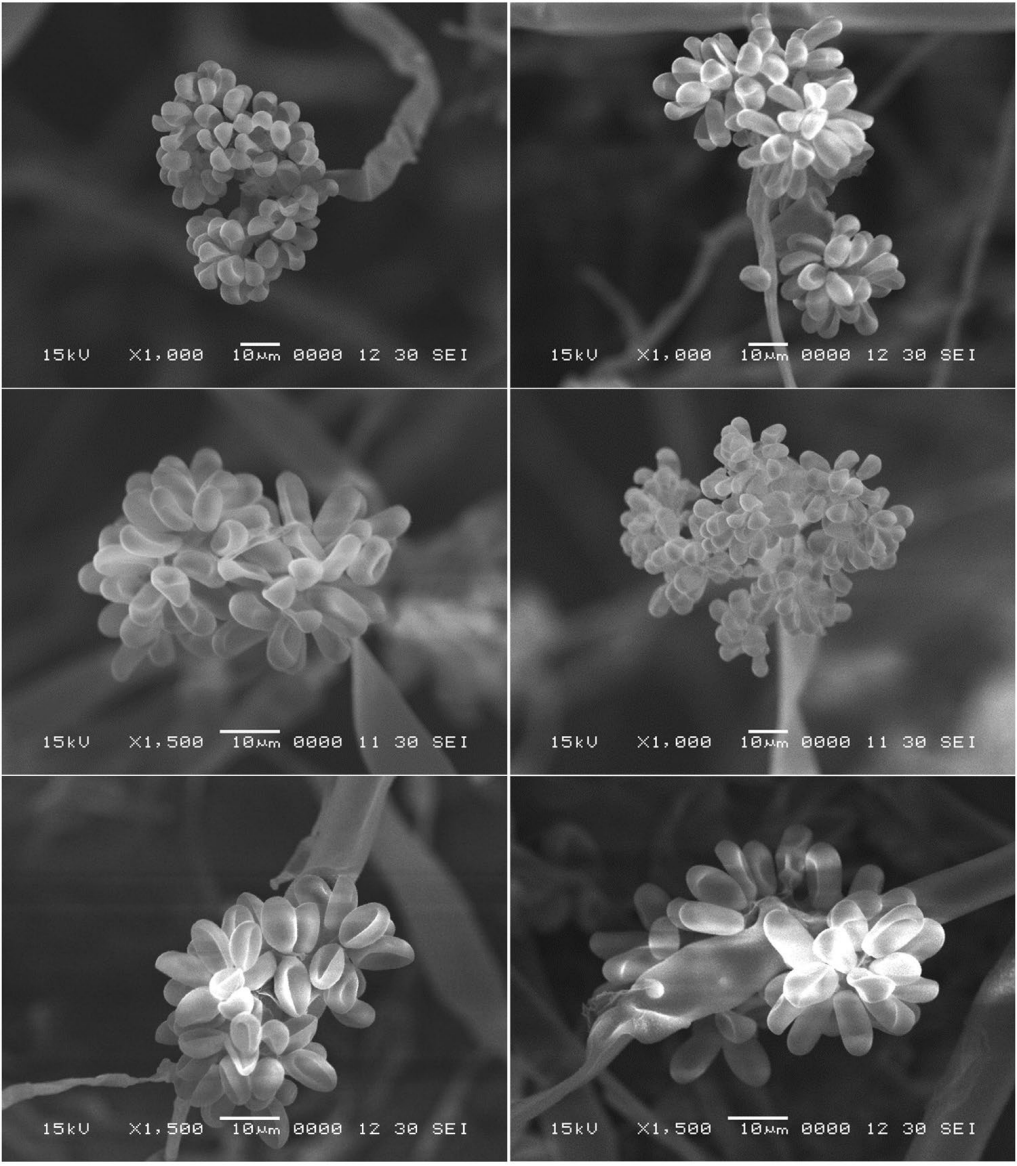
Scanning electron micrographs of *B. cinerea* SIB-1, showing conidial clusters carried on conidiophores at a magnification of 1000 × and 1500 × in different fields.

Data extracted from the SEM investigation (Fig. 8) show the conidial clusters carried on conidiophores and the branched mycelium, with conidiophores arising directly from the mycelium. They were more or less straight, monopodial branched toward the apex. The conidia were solitary and arranged in clusters or masses. The forms of the conidia observed were ellipsoidal and globose, and they were smooth, often with a slightly protuberant hilum and unicellular. The conidia measured 13.97 ± 2.04 μm in length and 9.53 ± 1.45 in width.

### Molecular identification

**Figure 9:**
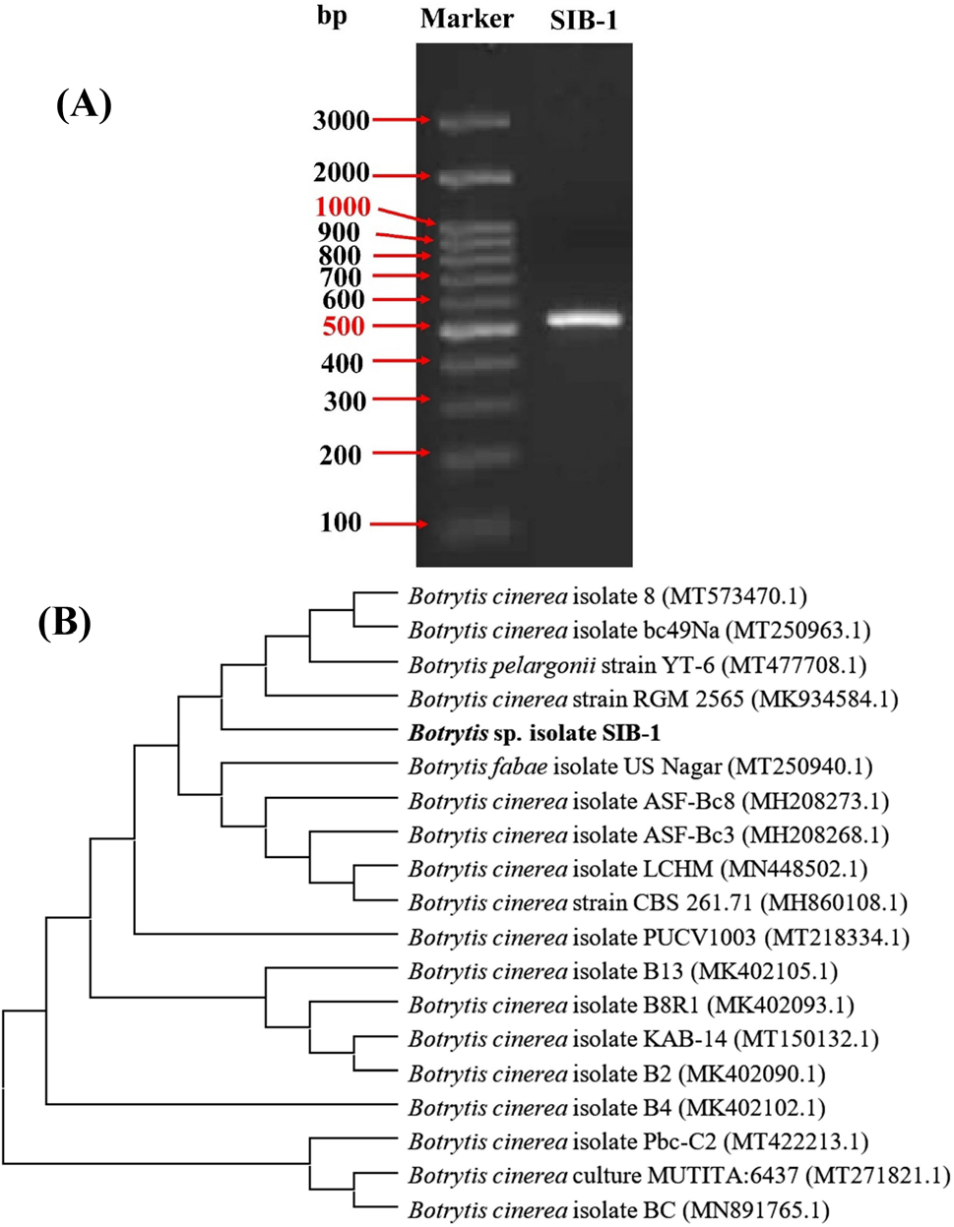
Profile of agarose gel electrophoresis of the PCR band of the PCR product of the amplified ITS fragment isolated from *B. cinerea* SIB-1 (**A**), and the evolutionary tree of the ITS gene's partial sequence of *B. cinerea* SIB-1 (accession number MZ570270), concerning the closely related sequences in GenBank (**B**).

A 500 bp fragment (Fig. 9A) of the internal transcribed spacer (ITS) region was amplified from genomic DNA of *B. cinerea* isolate SIB-1 by using a specific primer. (Supplementary Firgure. S1) The amplicon was sequenced and compared to the NCBI public genome map using the BLAST program. Phylogenetic analysis using the neighbor-joining method was performed (Fig. 9B) to determine the relatedness between the sequenced gene and its homologs in other organisms. The created phylogeny is totally annotated and displays a tight correlation with those of comparable strains. Multiple nucleotide sequence alignment showed that the *B. cinerea* SIB-1 isolate was closely related to *B. cinerea* strain RGM 2565 (K934584). Accordingly, the current strain was banked in the NCBI GenBank with the accession number MZ570270.

### Antifungal activity of CNPs

**Figure 10:**
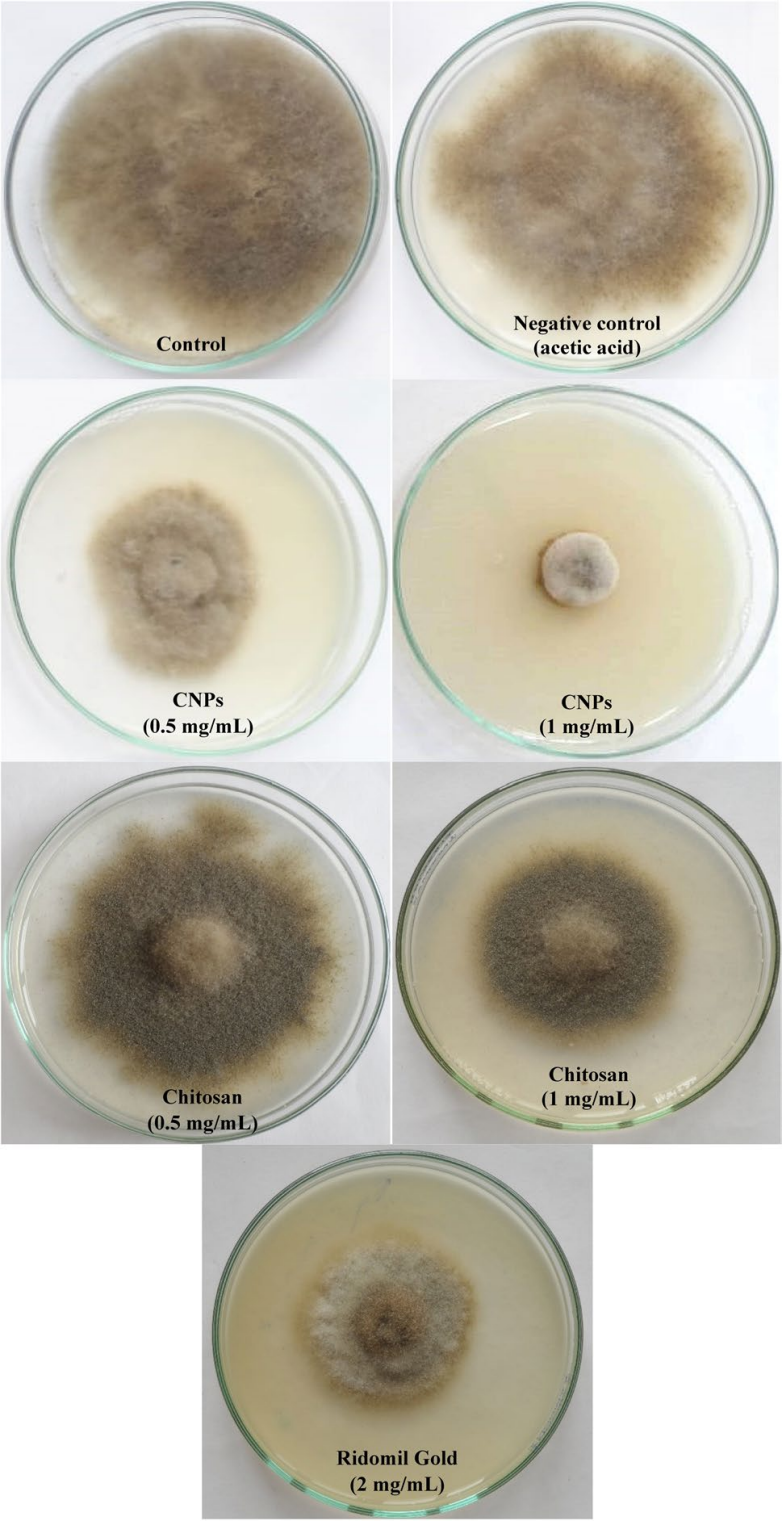
Antifungal activity of CNPs produced using *Pelargonium graveolens* leaf extract against *B. cinerea* SIB-1*.*

The in vitro antifungal activity of CNPs and chitosan (Fig. 10 and Table 4) revealed an obvious reduction in the growth of *B. cinerea* SIB-1 on potato dextrose agar plates, but the greatest fungal inhibition was the result of CNPs treatments. CNPs inhibited fungal growth by 73.58% at 1.0 mg/mL, while chitosan showed only 31.16% growth inhibition under the same condition. In comparison, the recommended dose of Ridomil Gold (positive control) caused about 50% inhibition of the fungal growth.

**Table 4:** Percentage of mycelia inhibition of *B. cinerea* SIB-1 by CNPs and chitosan after 10 days of incubation at 28 °C.

Concentrations (mg/mL)	Inhibition of fungal growth, %	
	CNPs	Chitosan
0.0	0.0 ± 0.00^e^	
0.5	44.15 ± 3.43^b^	11.25 ± 2.75^d^
1.0	73.58 ± 0.75^a^	31.16 ± 3.89^c^

Means followed by the same letter are not significantly different based on Duncan’s multiple range test at 5%.

**Figure 11:**
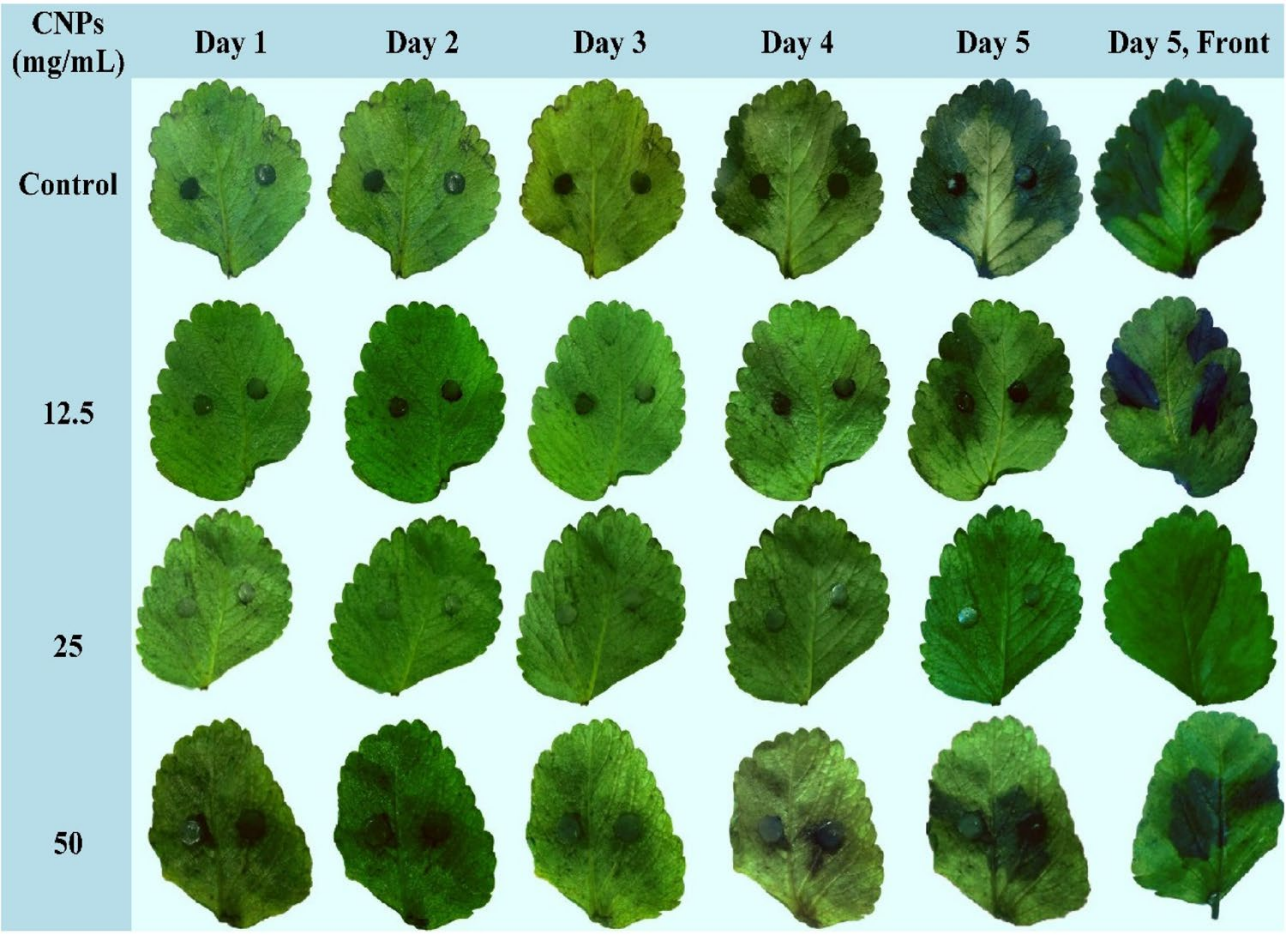
The protective effect of chitosan nanoparticles against gray mold disease caused by *B. cinerea* SIB-1 on detached strawberry leaves using different CNPs concentrations.

To investigate the protective roles of CNPs against gray mold disease developed by *B. cinerea* SIB-1, different concentrations of the CNPs were tested on detached strawberry leaves (Fig. 11). The average percent of leaf area infected was determined for each concentration. In general, after five days, leaves treated with the CNPs had a lower disease severity compared with the control, which had a mean infected area of 85% with a severity class value of 4. The lowest disease severity (class 0) was observed at the concentration of 25 mg/mL, which only showed a 3% infected area. However, CNPs at 12.5 and 50 mg/L recorded leaf area infections of 27 and 23% with severity class values of 2 and 1, respectively. It was found that the disease dramatically progressed over time from one to five days on both the adaxial and abaxial sides.

## Discussion

Nanotechnology has had a significant impact on several high-tech businesses in recent years, and it has been demonstrated that it has an impact on many microbial species, as well^1^.

There are several advantages of nanoparticles over the bulk form. Biologically, owing to the tiny size, nanoparticles straightforwardly penetrate and are easily taken up by the cell, which permits proficient accretion at the target site in the organism. Moreover, the retention of the nanoparticles at the target site has a longer clearance time, leading to an increase in therapeutic stability, bioavailability, and efficiency compared to the same dosage of the non-nanoparticulate form^7,18^.

Nanoparticles can be generated utilizing an assortment of strategies, including physical (ball milling, ultrathin films, spray pyrolysis, thermal evaporation, plasma arcing, lithographic procedures, pulsed laser desorption, layer-by-layer growth, sputter deposition, molecular beam epitasis, and diffusion flame synthesis of nanoparticles) and chemical (chemical solution deposition, electrodeposition, chemical vapor deposition, sol–gel process, soft chemical method, catalytic route, wet chemical procedure, hydrolysis coprecipitation, and Langmuir–Blodgett) methods, as well as hybrid techniques. These methods use high radiation and highly concentrated reductants and stabilizing agents that are destructive to human health and the environment as well^7,18–20^.

Alternatively, the biological-based procedure or green manufacture of nanoparticles is a bioreduction process with lower energy requirements. The technique is environmentally friendly and nontoxic, with greater stability, and nanoparticles are biosynthesized by applying a single-step process^18^. Moreover, to the best of the authors’ knowledge, all phytofabrication of nanoparticles is mostly restricted to metal ions, and no previous reports on CNPs phytofabrication.Many examinations have demonstrated that plant extracts act as potential precursors for the biosynthesis of nanoparticles in nondangerous manners. Therefore, plants are utilized effectively and economically in the biosynthesis of several metal-nanoparticles^7,10^.

The universal procedure of metallic nanoparticle biosynthesis employs the plant as a bioreducing agent and metallic salt as a precursor, resulting in biocompatible and stable nanoparticles. This promising route of nanoparticle production using a biological system utilizes three main approaches, i.e., 1) the selection of solvent intermediate, 2) the choice of an ecological, benign reducing agent, and the choice of a nontoxic material as a capping agent to stabilize the biosynthesized nanomaterials^21,22^. Additionally, twelve well-known green chemistry principles have now become a reference guide for developing less hazardous chemical products^23^.

All previous approaches were found to be achieved in the selected plant (*Pelargonium graveolens*) and the transformed polymer (chitosan), additionally, the 12-green chemistry principles were strongly applied in the current work. To the best of the authors’ knowledge, no previous work has reported on the synthesis of chitosan polymers into nanoforms using *Pelargonium graveolens* plants. Accordingly, the current paper describes a novel protocol for the green biosynthesis of CNPs, employing the leaf extract of *Pelargonium graveolens.* This procedure offers several merits over ordinary fabrication procedures.

Before proceeding to maximize the biosynthesis of CNPs, a primary characterization test based on UV/visible spectra was applied to ensure the development of the nanoparticles. The current absorption peak wavelength was detected at 295 nm; this result is in harmony with that previously reported at 285 nm^24^ and 320 nm in the UV region^2^. Compared with CNPs, the UV/visible spectrum of chitosan showed a wider absorption band intensity; therefore, the sharp intensity level of the CNP biopolymer indicates the success of the phytofabrication of CNPs^2,24^.

Exploring the experimental data of CNPs of the design matrix shows the highest level of CNPs located at the middle levels of the three tested variables, indicating the accuracy of both selected independent variables and their tested levels. It was obvious that the predicted values of CNPs were very adjacent to those of the trial CNPs; consequently, the residuals or error values were at their minimum, signifying another proven accuracy of the investigated parameters and their levels.

For the selection of the most appropriate model, the effect of each model term was screened at the *P* level of 0.05. The tested terms (factors) that had lower *P* values were considered significant and reliable for the modeling process. Regarding the model selection, R^2^ is considered a very important selection criterion; if R^2^ is higher than 0.9, the regression model is defined as very significant, and the model is adequate; however, the R^2^ value should not be lower than 0.75^25^. The current quadratic model had high R^2^, adjusted R^2^, and predicted R^2^ values, which were very close to one. Consequently, the quadratic model was the best-fitted model.

R^2^ is defined as the amount of change in the observed (experimental) response (CNPs) that is described by the three tested factors. Generally, R^2^ can help choose the best-fitted model. All types of R^2^ range from zero to 1. The closer to 1, the better the modeling of the experimental data. Interestingly, increasing the number of factors (predictors) leads to a continuous increase in the R^2^ value, irrespective of the significance of the factors. Therefore, the adjusted R^2^ is an improved R^2^ that considers the number of factors (variables) in the model. Contrary to R^2^, the adjusted R^2^ may be reduced with the addition of extra terms (factors) to the model. Therefore, the adjusted R^2^ is a better indicator than R^2^ to judge how well the model fits the data. The predicted R^2^ is used to determine the degree of the predictive capability of the model, e.g., to predict the value of the CNP response at new levels of the tested factors. Moreover, it is more beneficial than the adjusted R^2^ for comparing and selecting the models. Therefore, the quadratic model was selected as a modeling base in CNP bioprocessing.

The BBD data were subjected to ANOVA. The model exhibited a high *F*-value and low *P*-value; additionally, the lack-of-fit was not nonsignificant, indicating the significance of the proposed overall model. Moreover, the ratio of adequate precision is higher than 4, which is a suitable indicator that this model can be successfully employed to work within the tested range of the various tested factors along with the design space to maximize CNP biosynthesis. Another precision and trustiness of the experimental design can be noted by the lower value of the C.V. and greater value of adequate precision, which are desirable for the reliability of the model.

The weight of every individual factor was diagnosed, and the *P*-value was again utilized. However, the values of *P* indicate that the model terms are significant (< 0.05), indicating that they are important phytofabrication parameters of CNPs. This also suggests that the variables and their established levels, as well as the investigational design, are well defined and attain the peak performance of CNP phytofabrication. Therefore, the projection model was created based on such proven terms.

Data of ANOVA displays that the predicted R^2^ and adjusted R^2^ are close to each other. The values of both kinds of R^2^ should be less than 20% of each other to be in decent agreement^25^. In the current investigation, the predicted R^2^ was in harmony with the adjusted R^2^ value, indicating high compatibility between the predicted and experimental values of CNP biosynthesis and indicating the satisfactory predictive ability of the model within the examined range.

Some of the model terms showed a negative coefficient estimate value, which indicates that such a variable has an antagonistic effect on CNP biosynthesis by *P. graveolens* at higher concentrations. The positive coefficient value, on the other hand, indicates a cooperative effect, and the variable(s) increase CNP biosynthesis with the continuous increment of the level of the investigated factor within the region of the experiment.

Model adequacy was checked. The normal plot of residuals was plotted to check the externally studentized residuals versus normal probability (%). Values show an equal distribution of the residual data, indicating that the variance of CNP biosynthesis was independent of the biosynthesis process, thus supporting the adequacy of the model. Moreover, residuals were found to be very low at all tested points. This implies that the model can fit the actual experimental data faithfully. Additionally, the model prediction points vis the actual points lie much nearer to the line of perfect prediction. Thus, the model has a significant generalization capacity for CNP biosynthesis. Moreover, the Box-Cox plot of the model transformation of chitosan nanoparticle biosynthesis using *P. graveolens* leaf extract confirms the suitability of the design and data. The value of l concluded no recommendation for data transformation in this model. Consequently, these two adequacy tests authorize the aptness of the design and obtained data. Model adequacy was checked by plotting the normal probability of the externally studentized residuals. Most of the data points aggregated thoroughly around the straight line and were, thus, considered normally distributed without linearity. No value is located away from the general mean since the extreme residual values on both sides are not wanted. The plot of predicted vis actual values. The pattern also displays a normal distribution, supporting the adequacy of the model.

Analysis of the 3D plots demonstrates that all the pair tested factors generated a peak of CNP biosynthesis around the center point of the design space, meaning that the tested ranges of the three factors were carefully selected, and the model best fit the design.

To find out the best-predicted combinations that maximize CNPs, the desirability function was used, whose value ranges from undesirable (zero) to desirable (one). As the response approaches the goal, the desirability value becomes closer to 1. The desirability value is generally estimated as a mathematical evaluation of the optimization process before experimental validation^26^. Accordingly, the optimum levels of the incubation period, temperature, and initial chitosan concentration that maximize CNP biosynthesis by *P. graveolens* were estimated by solving the prediction equation. Among several options, the best solution was selected based on the desirability value. The current desirability value was sufficiently high since it reached the peak that validate the optimization process. Then, the predicted amount of CNPs was estimated, which was found to have a high intensity of agreement between the experimental values, suggesting that the desirability function effectively ascertains the best-predicted situations for the green synthesis of CNPs by using *P. graveolens* leaf extract.

The theoretical estimation of the polynomial model is an estimate based on a reasonably studied area of the tested independent variables; therefore, the guarantee of the real prediction effectiveness of the equation under real conditions is critical. However, the theoretical value of CNP biosynthesis was valid, since it showed close similarity to that of the experimental one. That is, in turn, produces strong evidence for the fitness of the design and the modeling process, utilizing the tested ranges of the studied variables.

Following the optimization conditions of the biofabrication process of CNPs from chitosan by *P. graveolens* leaf extract, the surface morphological structure of the obtained CNPs was monitored by SEM, which are widely considered the main accepted procedures for the characterization of nanoparticles. These techniques are erroneously used interchangeably, but in reality, they vary substantially. However, both techniques provide some similarities but a distinct analysis, which is why the accurate interpretation of their images is essential. Collectively, SEM and TEM offer powerful tools for the investigation of size, shape, surface area, crystal structure, and morphological structure. Although TEM systems can bring much greater 2D resolution for size analysis, SEM provides accurate information about the 3D surface and shape features^27,28^.

The 3D SEM image of CNPs exhibits a good dispersion of the nanoparticles, which are entangled to form a larger exposed surface area, making the CNPs very appropriate for adsorption^29^. Like the current phytofabricated CNPs, most of the CNPs prepared from chitosan were spherical in shape^30^, and few had oval pleated^31^ or rod-shaped structure^29,32^.

The 2D TEM image undoubtedly indicates that the CNPs show a highly porous surface owing to low agglomeration attributes. These porous and agglomerated CNPs have been considered key phenomena for the synthesis of novel CNPs, hence maximizing their usefulness as antibiological phytosynthesized nanomaterials in biomedical and agricultural applications, where the porous nature can effectively adsorb harmful chemicals and antagonize the pathogens^2,33^. In contrast to bulk materials, which have lower porosity, nanoparticles with high porosity have a greater specific exterior area and high reaction activity^1^.

The terms agglomeration and aggregation are repeatedly used interchangeably, but they definitely differ, where agglomeration indicates more weakly bonded particles and aggregation indicates strongly bonded or fused particles. In our case, the CNPs showed low agglomeration without aggregation, and the low agglomeration phenomenon is accepted since many nanoparticle types have high ionic strength and agglomerate in aqueous matrices, such as in phosphate-buffered saline and cell culture medium^34,35^.

EDXS study was used together with electron microscopy investigation to analyze the component elements of CNPs. When the electron beam of SEM hits the inner shell of an element atom, its inner-shell electron is relocated by another electron from an outer shell to fill the vacancy, and the process is accompanied by the release of an energy difference in the form of an X-ray that is unique to the specific element. Moreover, the intensity of the specific X-ray is directly related to the concentration of the element in the particles^36^. However, the test confirms the presence of the various elemental compositions of native chitosan, confirming the uniformity and stability of CNPs during the biotransformation process.

The ζ-potential is an indicator of the stability of colloidal dispersions. The weight of the ζ-potential specifies the degree of electrostatic repulsion among similarly charged adjacent particles^37^. For tiny particles and molecules, a high ζ-potential confers stability and resists aggregation of nanoparticles in the solution or dispersion. In contrast, at small ζ-potentials, attractive forces may exceed, leading to flocculation owing to the breakdown of the dispersion. Therefore, colloids with high negative or positive ζ-potentials are more electrically stabilized than those with low ζ-potentials, which tend to coagulate or flocculate^38^. The current ζ-potential value of CNPs suggests nanoparticles with good stability. The CNPs were positively charged. From an antimicrobial point of view, when ζ-potential is positive, particles can easily interact with the negatively charged cell membrane and/or DNA of a biological system and can then be released simply into the cytoplasm of the cell^39^.

Regarding FTIR, the presence of various intense bands indicates the presence of a capping agent, which acts as a stabilizer that inhibits the overgrowth of nanoparticles and prevents their aggregation and/or coagulation in colloidal synthesis. Therefore, the observed intense bands were matched with standard values to classify the functional groups. The first range of bands that appeared in the spectra was due to stretching vibrations of OH groups at wavenumbers ranging from 3736 to 3442 cm^−1^, indicating the presence of alcohols and phenols. The stretching vibration of methylene (C=H) was at 2350 cm^ −1^. It is also already known that the band at 2350 cm^−1^ generally arises from the background CO_2_^40^ and has no corresponding group associated with the chitosan structure. Absorption at wavenumber 1572 was correlated to the vibrations of carbonyl bonds (C=O stretching) of the amide group CONHR or protonated amine (NH_2_. Bending vibrations of the methyl group (C-H bending, alkane) of CNPs were visible at 1413 cm^−1^. Absorption in the wavenumber range 1072 and 914 cm^−1^ is generated from the stretch vibration of CO groups (COH and COC) in the oxygen bridge, emerging from the deacetylation of chitosan. At the end of the FTIR spectra, the small peak at 645 cm^−1^ corresponds to the wagging of the saccharide structure of chitosan^41^. FTIR analysis strongly emphasizes the structural stability of chitosan during phytoconversion into CNPs.

XRD analysis is a fast practice, primarily used in materials science for the phase identification of a crystalline nature and can deliver information on unit cell dimensions; thus, the XRD pattern is considered the fingerprint of periodic atomic arrangements in a given material^32^. The XRD of the current CNPs showed three peaks at 2-theta of 13, 19, and 35°. It is conventionally accepted that chitosan stretches two characteristic peaks at 2-theta of 10 and 20°, and the current shift that occurred to CNPs from the normal chitosan peaks indicates the amplified amorphous nature, thus lessening the crystal structure of chitosan, which comes in line with studies that focused on decreasing the crystallinity for improving the sorption properties of the materials^32,42^.

Both TGA and DSC investigations were performed, and both are measures of the thermo analytical features used to describe the analysis of nanoparticles that take part in chemical reactions over a controlled temperature range. TGA measures the differential thermal analysis in terms of the change in mass of the sample in relation to temperature changes or as a response of time with constant temperature and/or constant mass loss. DSC, on the other hand, measures the heat flow released or required against the temperature change at a particular time. The main dissimilarity between TGA and DSC is the method of measuring the changes in samples that are triggered by heat^29,31,32,43^.

At the beginning of TGA, common drying as a function of temperature can easily cause a quick initial drop in CNP mass due to the loss of residual water bound to the two polar groups in CNPs, which is not known to correspond to any chemical reactions^32^. Another reason for the drop in weight at the beginning step may be due to the dehydration of the saccharide rings, depolymerization, and decomposition of volatile products^29^. Next, successive weight losses in CNPs with increasing temperature may be due to evaporation and/or sublimation; however, multistage decomposition shown as a step-like pattern is due to the thermal degradation of CNPs^43^.

The loss of weight in the next stages of thermal analysis may be due to the decomposition of the polymer matrix; however, the CNPs did not fully decompose at the high temperature (800 °C) and conversely showed some stability in the polysaccharide structure. This result indicates that CNPs are thermally stable over a temperature up to 800 °C, which may be due to the high crosslinking of the CNPs that forms a stronger and stiffer hydrogel network^29,32,43^.

However, the thermal analysis steps were not blended during dynamic TGA; however, there is still a possibility of hidden interference of the decomposition steps, necessitating either far slower heating rates or stepwise TGA methods. That is why TGA itself may not be sufficient to identify the decomposition products, therefore chemical testing such as DSC is often, required alongside TGA, to ascertain the identities of suspected decomposition products^31^.

DSC of CNPs was performed to highlight phase transitions and clarify every single step of the thermal degradation mechanism. Usually, the temperature program for a DSC analysis is designed such that the sample holder temperature increases linearly as a function of time. That, in turn, can offer pieces of evidence about physical phenomena, such as glass transition, thermal stability, and purity^31^. Two definite broad endothermic peaks were generated, the first at a lower temperature that was due to the removal of absorbed water. The second endothermic peak that appeared at 242 °C is generally associated with the breakage of cross-linkage of CNPs. Additionally, the higher value of the glass transition temperature is due to the presence of a crosslinking agent and high thermal stability. Only a single glass transition in the DSC heating curves indicates the uniformity of the CNPs under high temperatures^29^. The transformation of chitosan into CNPs decreases the crystallinity due to changes in the solid-state structure of chitosan due to crosslinking, and thus, the decomposition of CNPs occurs above 300°C^32^.

Interestingly, in the present study, all proceeding investigations came in harmony with each other to provide an accurate perspective for the characterization of CNPs. Moreover, the currently proposed phytofabrication method for CNP preparation is considered ideal for generating high-quality CNPs.

The developed phytosynthesized CNPs were monitored regarding their antifungal properties. Depending on the molecular weight, concentration, degree of substitution, and the type of functional groups on chitosan, the free chitosan polymer exhibits various antifungal activities against a wide array of fungi. Derivatives of the polymer can be formed to target specific pathogens. Chitosan shows natural antifungal capability without the necessity for any chemical alterations^2^, which is why the generated CNPs were tested against the isolated *B. cinerea* SIB-1.

Koch's postulates were applied to the isolated fungi to confirm the pathogenic ability and to select the most aggressive isolate as well. The colony characteristics of the isolated fungus and SEM investigation showed the typical features of the already known phytopathogenic *B. cinerea* SIB-1. These features are in line with those previously described^17^.

A severe phytopathogen (*Botrytis cinerea* SIB-1) was used in this study as a model for the evaluation of CNPs as an anti-biological agent. The main reason for selecting such phytopathogen is the wide host range since it can infect more than 200 host plants, and it can infect several parts of the plants, including the upper parts such as seeds, leaves, bulbs, and other propagation material at pre- and postharvest stages. Moreover, *Botrytis* spp. infect the host plant in all climate areas of the world and under great humidity in the presence or absence of water films. The fungus can generate high numbers of conidia that pose a long-lasting threat to susceptible hosts; in addition, the genotypic and phenotypic variation of the fungus is another broad-spectrum thread for the plant production sector^16,17^. Importantly, the fast alterations in populations and resistance in response to exposure to xenobiotics, e.g., fungicides, are quite widespread in the genus^44^, urging the discovery of alternative commercial approaches of considerable disease suppression to be integrated into crop management protocols^15^.

The selected fungus was identified as *B. cinerea* SIB-1 on a molecular basis, which is sensitive and specific for the rapid recognition of filamentous fungi at different systematic levels. The ITS region is usually used and can be adequate for fungal identification at the species level. The ITS region is also contemplated among the markers with the fastest and uppermost probability of precise identifications for a very broad group of fungi^45^. Interestingly, the culture morphology, SEM investigation, and molecular identification computably confirmed the fungus to be *B. cinerea* SIB-1.

Gray mold caused by the phytopathogenic *Botrytis cinerea*, is a serious disease that affects all strawberry growing regions and is the main cause of concern most years. The gray mold disease is a problem not only in the field, but also during storage, transportation, and marketing of strawberries as a result of severe rot as the fruits begin to ripen. Leafs, fruit caps, flower stalks, petals, and crowns are among the other parts infected by the fungus. CNPs showed strong inhibition against *B. cinerea* SIB-1. Possible protocols include inhibition of mycelial growth and sporogenesis^3^.

There are three mechanisms proposed as the inhibition mode of chitosan. In the first mechanism, the cell membrane of fungi is the main target of chitosan. The inhibitory effect of chitosan may be related to its interaction with the cell membrane of the fungal cell and alteration of membrane permeability^46^. The positive charge of chitosan allows it to interact with phospholipid components of the fungal cell membrane that are negatively charged. This increases membrane permeability, allowing cellular contents to leak out, ultimately resulting in cell death. The second mechanism involves chitosan acting as a chelating agent, binding to trace elements and rendering them unavailable to fungi for normal growth. Finally, the third mechanism proposed that chitosan could pass through fungi's cell walls and bind to their DNA or proteins. This will stop the production of essential proteins and enzymes by inhibiting the synthesis of mRNA^3^. Chitosan inhibited mycelia growth, sporulation, and spore germination. It induces morphological changes characterized by excessive branching, mycelial swelling, agglomeration of hyphae, abnormal shapes, dissolution of protoplasm, large vesicles, cytoplasm aggregation, or empty cells devoid of cytoplasm in the mycelium^47^.

Ridomil Gold is a systematic fungicide that inhibits fungal development by interfering with the biosynthesis of sterols in the cell membrane. Thus, provides excellent disease control, ensures double protection to the target plants due to systemic activity of Mefenoxam fungicide and contact protective activity of mancozeb fungicide. Mefenoxam penetrates rapidly into the plant through the leaves and stems and is distributed upwards with the flow of sap. This way, new growth is protected as well. Mancozeb provides a protective film on the surface of the plant and inhibits germination of the spores and controls leaf and tuber blight as well as leaf spot disease.

The current experimental results on strawberry leaves show that treatment with CNPs reduced infection lesions. A key factor for a pathogenic fungus to be able to magnificently infect plants is to secrete a category of effector proteins into plant cells, which makes plants more susceptible to diseases^48^. These effector proteins decreased after treatment with chitosan^5^; moreover, chitosan can stimulate defense-related enzymes and augment the accumulation of antimicrobial ingredients in the infected plant, mainly diminishing the success rate of the fungal infection and inducing plant resistance^49^.

It is of essential importance to note that leaf treatment with a high concentration of CNPs (50 mg/mL) was found to have a reverse effect on the infected area compared with those of lower concentrations. These results suggested that increasing the concentration of nanoparticles might result in the crowding of these particles on the leaf pores that limit their penetration into the inner tissues. Furthermore, several findings concluded the potential phytotoxicity of these nanoparticles at high concentrations. In this respect, the application of CNPs at a concentration higher than the optimum causes a reduction in the mineral and nitrogen contents of the coffee leaves^50^. Similarly, high concentrations of chitosan nanoparticles markedly reduce the growth and development of *Capsicum annuum*, while lower concentrations have a growth-promoting effect^51^. These results suggest additional investigation on the optimum concentration of CNPs and further indicate the need for caution when using CNPs to reduce the harmful side effects of elevated concentrations.

## Materials and methods

### Formulation of plant extract

Fresh leaves of *Pelargonium graveolens* were collected from the El-Natrun area (30° 22′ 39″ N latitude and 30° 21′ 1.08″ E longitude), Northern West Nile Delta, 62 miles from Cairo, Egypt. The leaves were washed thoroughly three times with tap water and then with distilled water to eliminate any impurities. Finally, 25 g of thoroughly washed and finely cut leaves were dipped into a conical flask containing 100 mL of distilled water, mixed, and boiled for 10 min. After boiling, the solution was then filtered through filter paper (Whatman No. 1). The filtered extract of *Pelargonium graveolens* was collected and used for the biosynthesis of CNPs.

### Phyto-synthesis procedure of CNPs

Chitosan, obtained from Bio Basic Inc., Toronto, Canada, was liquefied at 1% (w/v) with acetic acid (1%, v/v), and the pH was raised to 5 with 1 N NaOH and kept under magnetic stirring for 24 h. Equal amounts (10 mL) from each of the plant extracts and chitosan solution were blended and incubated at 50 °C under shaking for 30 min. After incubation, the turbidity was centrifuged at 10,000×*g* for 10 min. To remove the unreacted chitosan from the produced nanoparticles, it was washed several times with acetic acid solution. The precipitate was then obtained by centrifugation of the mixture at 10,000×*g* for 10 min. The resultant CNPs were then freeze-dried. The resultant CNPs were redissolved again in 1% acetic acid, and the UV/visible absorbance spectrum of the prepared CNPs was determined by a double beam spectrophotometer at 295 nm. A standard calibration curve prepared from known concentrations of CNPs was prepared in 1% acetic acid at various serial dilutions and was used to estimate the final concentration of CNPs (mg/mL).

### UV–Visible spectrum

The generated CNPs were monitored by detecting the peak absorbance at an array between 200–400 nm utilizing an Optizen Pop-UV/Vis spectrophotometer.

### Optimization of CNPs biosynthesis by BBD

The matrix of BBD was generated to establish the best levels of the chosen process variables affecting chitosan nanoparticle biosynthesis using *P. graveolens* leaf extract and to study the individual and interaction effects between these variables. The factors were incubation period (X_1_, 30–90 min), temperature (X_2_, 40–60 °C), and chitosan concentration (X_3_, 0.5–1.5%). Each process variable ranges at three levels (− 1, 0, + 1), with five central points, resulting in a total of seventeen trials (Table 1). The linear, interaction, and quadratic influences of the three selected process variables affecting chitosan nanoparticle biosynthesis were determined to explore the relationship between chitosan nanoparticle biosynthesis (*Y*) and the optimum level of each of the tested variables. The next second-order polynomial function was applied:

1 $$Y={\beta }_{0}+{\sum }_{i}{\beta }_{i}{X}_{i}+{\sum }_{ii}{\beta }_{ii}{{X}_{i}}^{2}+{\sum }_{ij}{\beta }_{ij}{X}_{i}{X}_{j}$$

where *Y* is CNP biosynthesis using *P. graveolens* leaf extract, *X*_*i*_ is the process variable at the coded levels, *β*_*0*_ is the regression coefficient, *β*_*i*_ is the linear coefficient, *β*_*ij*_ is the interaction coefficient and *β*_*ii*_ is the quadratic coefficient.

The investigational design and statistical examination were conducted using the Windows edition of Design-Expert software (version 12, Stat-Ease, Minneapolis, USA). The STATISTICA version 8 program was used to draw 3D surface plots.

### Characterization of CNPs

#### SEM investigation

The size, construction, and morphology of the CNP samples were examined. After coating by a gold sputter coater (SPI-Module), samples were examined by SEM (model JEOL-JSM-IT200) at 20 kV at the Electron Microscope Unit, Faculty of Science, Alexandria University, Alexandria, Egypt.

#### TEM investigation

Another morphology investigation of CNPs was carried out by TEM. The samples were examined with a transmission electron microscope unit (TEM; JEM-2100 Plus, JEOL Ltd., Japan) at the Central Laboratory, City of Scientific Research and Technological Applications, Alexandria, Egypt.

#### EDXS examination

EDXS is commonly used to determine the elemental composition and characterization of a sample. The electron beam focused on a single nanoparticle by TEM through the program functions to obtain insight information about the CNPs under observation.

#### ζ-potential of the synthesized CNPs

The zeta (ζ) potential determines the surface charge properties of the chitosan nanoparticles. The ζ-potential of the CNPs was quantified utilizing Malvern analytical Zetasizer software Ver. 7.13 equipped with a laser Doppler and detected with phase analysis light scattering. The measurements were performed at 25 °C, taking samples in the liquid state^52^.

#### FTIR spectroscopy analysis

FTIR spectroscopy assessment was conducted to analyze the surface properties of the CNPs, which were ground with KBr pellets used for FTIR measurements. The FTIR spectrum of CNPS was measured using a Shimadzu FTIR-8400 S spectrophotometer in the range of 4500–500 cm^−1^ at a resolution of 1 cm^−1^.

#### XRD pattern

XRD is a crucial technique for determining the structural properties of nanoparticles. The XRD patterns were recorded using a diffractometer (Bruker D2 Phaser 2nd Gen). The X-ray source was operated with a Cu anode with a voltage of 30 kV and a current of 10 mA. Diffraction intensity was measured at 25.7 °C and a scanning rate of 2°/min for 2θ = 10–50^53^.

#### TGA of CNPs

The CNP sample was withered at 60 °C for 1 h and mounted in a platinum sample pan. TGA was accomplished using a thermoanalyzer of type 50-H. TGA was operated in the range from 25 to 800 °C, with a 20 mL min^-1^ flow rate, under a nitrogen atmosphere at an increment of 10 °C min^−1^. The chart was drawn as temperature versus weight loss percentage.

#### DSC analysis

DSC (60-A) was used to assess the CNP pyrolysis pattern. The CNP sample was dried at 60 °C for 1 h and mounted in an aluminum sample pan. The examination was operated under nitrogen atmosphere conditions with a heating rate of 10 °C min^−1^ and a flow rate of 30 mL min^-1^. The thermogram was generated between 25 to 500 °C. The initial breakdown temperature for the CNPs, as presented by thermogravimetric analysis, was chosen for the DSC upper limit. The graph was mapped as heat flow versus temperature.

#### Isolation and identification of the fungal pathogen

The model fungus used in this study was *B. cinerea,* a causative agent of gray mold. The fungus was isolated from blighted fruits of strawberry (*Fragaria virginiana*, var. Fortuna) collected from open fields in the Menoufia government (30°18′ 02″ N latitude 31° 00′ 30″ E longitude) during March 2021. The collected samples were kept at 25 °C and 90% relative humidity for 4 days in a glass box to facilitate fungal sporulation. The purified culture was obtained by a single-spore technique on PDA medium, purified, and then stored (4 °C) until further use. Primary morphological identification was carried out with the aid of the Miclea et al.^17^ method. One pathogenic isolate of *B. cinerea* SIB-1 was selected based on the pathogenicity test. The most severe isolate was selected, and further confirmational identification methods were used. The morphology of the *Botrytis cinerea* SIB-1 was examined on potato dextrose agar plates after 7 days at 30 °C. The gold-coated dehydrated specimen was examined at different magnifications with Analytical Scanning Electron Microscope Jeol JSM-6360 LA operating at 15 kV at the Central Laboratory, City of Scientific Research and Technological Applications, Alexandria, Egypt. Then, molecular identification was applied.

#### Molecular identification

The selected isolate was subjected to molecular identification. The genomic DNA of *B. cinerea* SIB-1 was extracted utilizing the cetyltrimethylammonium bromide (CTAB) technique^54^ with some alterations. Mycelia of the selected isolate were scraped from the surface of a 4-day-old plate, homogenized in prewarmed CTAB extraction buffer, and incubated at 60 °C for 30 min. Cell debris was detached by centrifugation at 12,000 rpm for 20 min, and the extracted DNA was purified from chromatin-associated protein by phenol/chloroform treatment. Then, DNA was precipitated by isopropanol, washed with 70% ethanol, and air-dried. The DNA pellet was redissolved in TE buffer and utilized as a template for PCR amplification.

Genomic DNA of *B. cinerea* SIB-1 was utilized as a template for PCR amplification of the ITS regions using primers ITS1 (5'-TCT GTA GGT GAA CCT GCG G-3') and ITS4 (5'-TCC TCC GCT TAT TGA TAT GC-3') described by White et al.^55^. The specific primers were obtained from Clinilab (Clinilab Analysis Co., Egypt). The reaction mixture (25 µl) contained 5 µl of Taq red buffer, 20 ng of template DNA, 10 pmol of each primer, 0.25 U of Taq polymerase (Bioline, UK). PCR cycling conditions were as follows: 95 °C for 2 min, 30 cycles of 95 °C for 30 s; 50 °C for 30 min; 72 °C for 1 min; and 1 cycle of 72 °C for 5 min. The PCR product was checked on a 1.5% agarose gel stained with 0.05 μg/mL ethidium bromide, and the target band was purified with a GF-1 PCR clean-up kit according to the manufacturer's guidelines.

The ITS sequence was computationally evaluated using the BLASTn program (http://www.ncbi.nlm.nih.gov/BLAST). Sequences were aligned using Align Sequences Nucleotide BLAST. The obtained sequence was banked in GenBank to attain strictly related sequences; then, the accession number of the fungal strain was established. The evolutionary relationship was inferred, and the phylogenetic tree was generated using MEGA 10 software.

#### Antifungal activity of CNPs

Two tests were performed to investigate the in vitro antifungal activity of CNPs against the gray mold pathogen *B. cinerea* SIB-1. The first test was performed on agar plates containing PDA medium. The pH of the culture medium was adjusted prior to autoclaving using 1 M NaOH to be 6.5. So that, after sterilization and addition of CNPs or chitosan, the pH falls within the specified range (found to range from 5 to 5.5).

Known amounts of CNPs were dissolved in acetic acid solution (1%) with stirring overnight under aseptic conditions. The nanomaterial was added to PDA to ultimate concentrations of 0.5 and 1.0 mg/mL. To prepare the fungal inoculum, *B. cinerea* SIB-1 mycelia were taken from a single colony and transferred to potato dextrose agar plates. The inoculated plates were incubated at 28 °C for 7 days. A fungal disk (5 mm) was taken from the active growing margin and aseptically transferred to the center of the plates. The inoculated plates were checked daily until the control (zero CNPs) treatment touched the edge of the plate.

The in vitro antifungal activity of chitosan against *B. cinerea* SIB-1 was also investigated. On the other hand, the antifungal activity has been compared with the commercial Ridomil Gold fungicide on the mycelium growth of *B. cinerea* SIB-1 under the same conditions. Ridomil Gold is a leading fungicide recommended for controlling many important diseases on many crops. It is a mixture of both systemic (Mefenoxam) and contact (Mancozeb) fungicides to control certain diseases.

The second test was carried out to evaluate the protective effect of CNPs against gray mold disease. CNPs were prepared by centrifugation at 10,000 rpm for 10 min and washed three times to remove any residue of acetic acid. Then, the washed CNPs were resuspended in sterile distilled water at concentrations ranging from 0 to 50 mg/mL. Healthy detached strawberry leaves were collected, and surface sterilized with 2% sodium hypochlorite for 3 min and 70% ethanol for 1 min, rinsed with sterile water, and finally dried on filter paper. The collection of plant material has complied with relevant institutional, national, and international guidelines and legislation. The surface-sterilized leaves were then immersed in the nanoparticle solutions (0, 12.5, 25, and 50 mg/mL) for 5 min and placed on moist paper towels in 9 cm Petri dishes with their adaxial surface up. Two mycelial plugs (5 mm) were acquired from the margin of a 5-day-old culture of *B. cinerea* SIB-1 and adhered to the treated leaves, with five leaves for each treatment. The dishes were sealed with plastic film and incubated at 25 °C in the dark for up to 5 days. The average percent of leaf area infected was determined for each concentration. Disease severity was evaluated in comparison to a disease severity scale of 0 (healthy leaf), 1 (< 25% infection); 2 = (25–50% infection) 3 = (51–75% infection), and 4 (> 75% infection)^56^.

## Conclusions and challenges

The current study introduced a novel biological-based approach for the green synthesis of chitosan nanoparticles using *Pelargonium graveolens*. Optimization of the bioprocess parameters was studied and maximized. All characterization tests on CNPs confirmed the suitability and efficacy of the plant as the bio-converter agent. The antifungal activity of the generated CNPs against a model phytopathogen with a wide host range (*B. cinerea* SIB-1) proved the ability of the CNPs to replace or minimize the extensive use of pesticides and to be applied in various technological and medical fields.

Although the data of the current study are promising, other several challenges are still recommended to be elucidated, such as the exact mode of action of the generated CNPs, the suitable inhibitory doses on a wider range of fungi, and the cytotoxicity of CNPs. However, the large-scale production of CNPs is another challenge before being approved as a commercial product.

## Supplementary Information

Supplementary Figures.
