# Supplementary material for: An innovative green synthesis approach of chitosan nanoparticles and their inhibitory activity against phytopathogenic Botrytis cinerea on strawberry leaves
Source: Sci Rep. 2022 Mar 3;12:3515. doi: 10.1038/s41598-022-07073-y (PMC8894456; doi:10.1038/s41598-022-07073-y)
Supplement: Supplementary file 1 — Supplementary Figures. [file 41598_2022_7073_MOESM1_ESM.docx]

**Original Article**

**An innovative green synthesis approach of chitosan nanoparticles and their inhibitory activity against phytopathogenic** ***Botrytis cinerea* on strawberry leaves**

**Noura El-Ahmady El-Naggar^1^; WesamEldin I.A. Saber^2^; Amal M. Zweil^3^; Shimaa I. Bashir^4^**

^1^ Department of Bioprocess Development, Genetic Engineering and Biotechnology Research Institute, City of Scientific Research and Technological Applications (SRTA-City), New Borg El‑Arab City 21934, Alexandria, Egypt. http://orcid.org/0000-0001-8493-9194

^2^ Microbial Activity Unit, Department of Microbiology, Soils, Water and Environment Research Institute, Agricultural Research Center, 12619, Giza, Egypt. https://orcid.org/0000-0003-0631-4089

^3^ Plant Biotechnology Department, Genetic Engineering and Biotechnology Research Institute, University of Sadat City, Egypt.

^4^ Department of Plant Protection and Biomolecular Diagnosis, Arid Land Cultivation Research Institute, City of Scientific Research and Technological Applications (SRTA-City), New Borg El‑Arab City 21934, Alexandria, Egypt.

**Correspondence should be addressed to:**

**Prof. Noura El-Ahmady Ali El-Naggar**

**Address:**

Bioprocess Development Department,

Genetic Engineering and Biotechnology Research Institute,

City of Scientific Research and Technological Applications,

New Borg El- Arab City, 21934, Alexandria, Egypt

**Tel:** (002)01003738444

**Fax:** (002)03 4593423

**E-mail:** nouraelahmady@yahoo.com

| 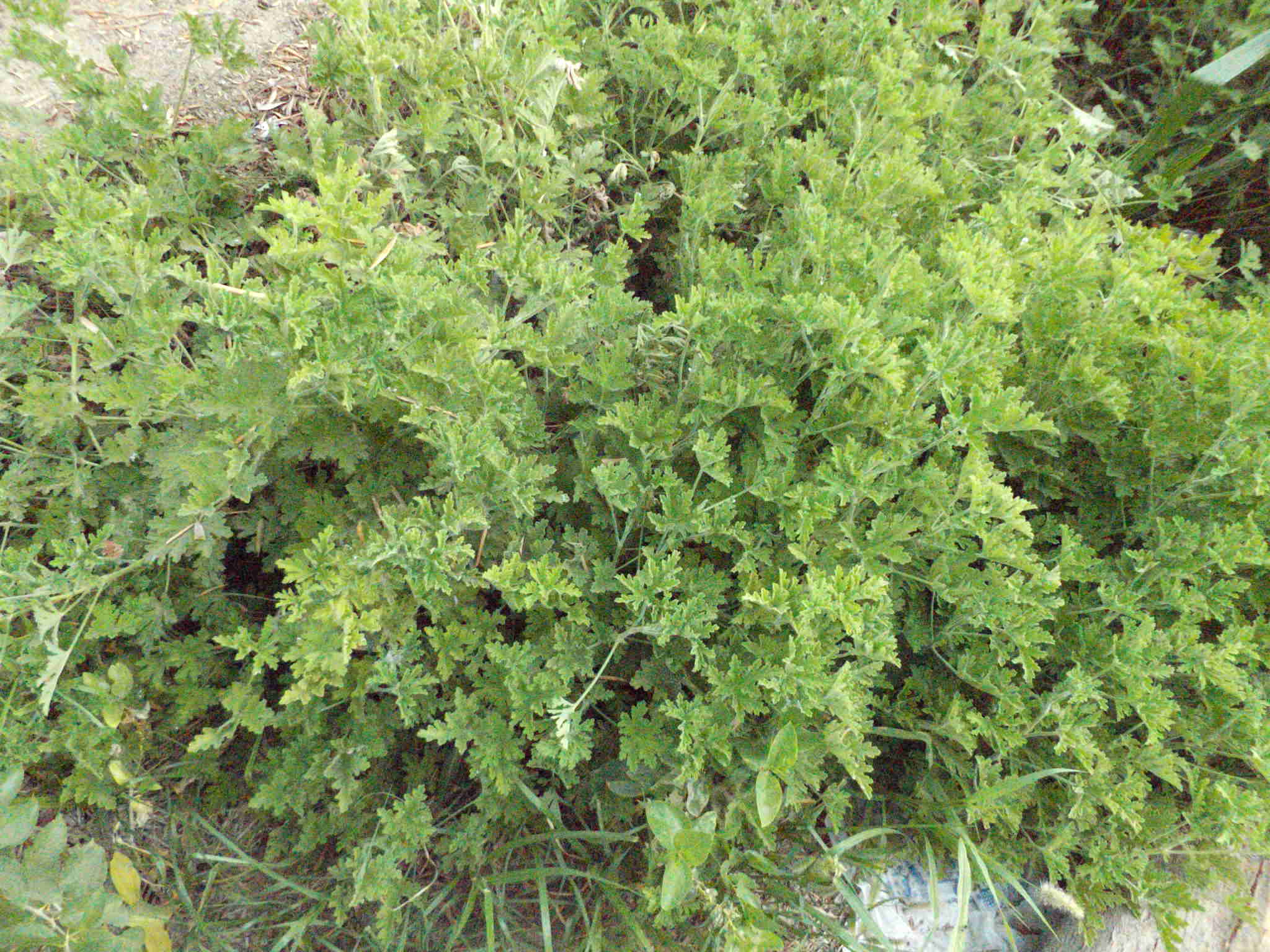 |
| --- |
| 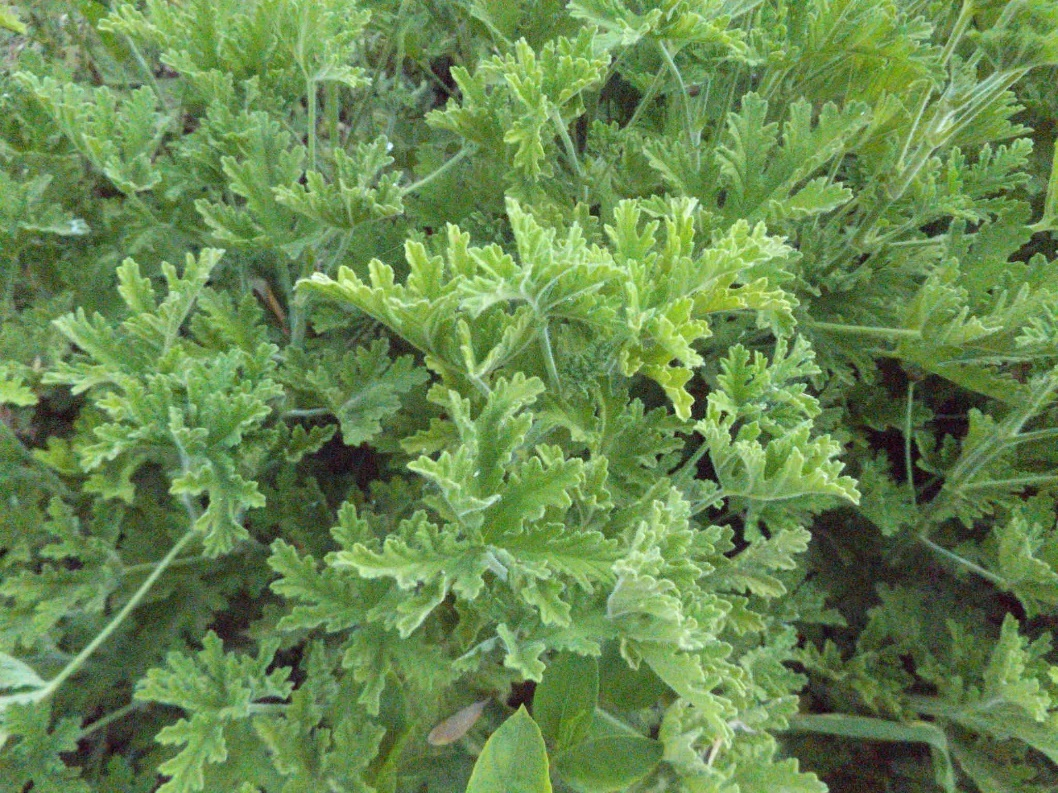 |

**Supplementary Figure 1**: General view of *Pelargonium graveolens*, showing the deeply incised, velvety, and soft to the touch leaves that are used in the current study.


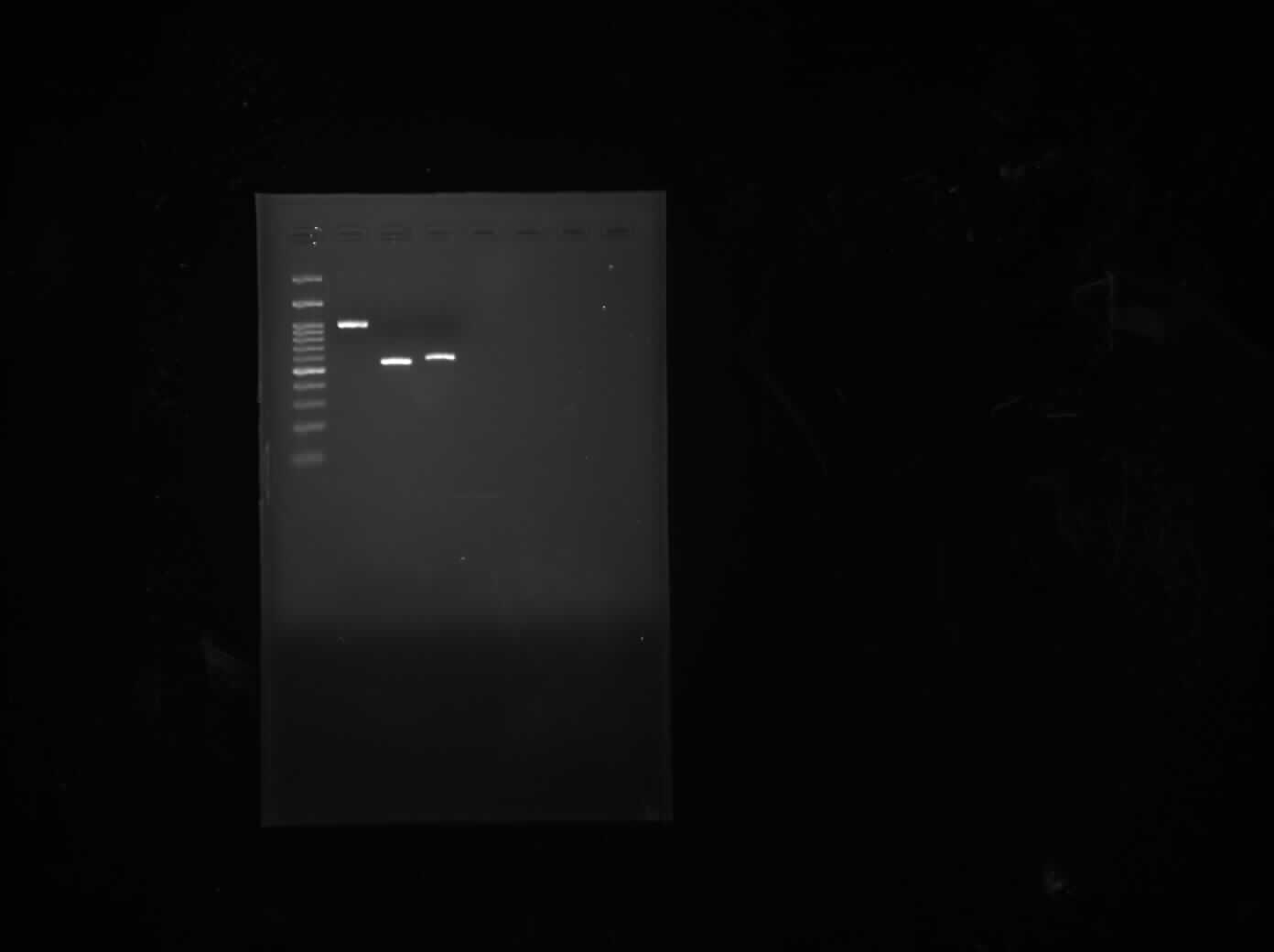


**Supplementary Figure 2**: Complete gel for Figure 9B.
